# Cycling of labile and recalcitrant carboxyl-rich alicyclic molecules and carbohydrates in Baffin Bay

**DOI:** 10.1038/s41467-024-53132-5

**Published:** 2024-10-09

**Authors:** Kayla McKee, Hussain Abdulla, Lauren O’Reilly, Brett D. Walker

**Affiliations:** 1 Department of Earth and Environmental Sciences, University of Ottawa, Ottawa, ON Canada; 2 Department of Physical and Environmental Science, Texas A&M University-Corpus Christi, Corpus Christi, TX US; 3 Department of Earth System Science, University of California, Irvine, CA US

**Keywords:** Carbon cycle, Marine chemistry, Marine chemistry

## Abstract

Marine dissolved organic matter (DOM) is an important, actively cycling carbon reservoir (662 GtC). However, the chemical structure and cycling of DOM within rapidly warming, polar environments remains largely unconstrained. Previous studies have shown rapid surface cycling of carbohydrates as biologically-labile DOM (LDOM). Conversely, carboxyl-rich alicyclic molecules (CRAM) are often used as examples of biologically-recalcitrant DOM (RDOM). Traditional DOM isolation methods (e.g., ultrafiltration (10–30% of DOM) and solid-phase extraction (40–60% of DOM) induce chemical-, size- and compositional-bias – complicating inferences to total DOM cycling. Here, we use a total DOM proton (^1^H) nuclear magnetic resonance (NMR) spectroscopy method to show carbohydrates and CRAM have high concentrations in the surface ocean and low concentrations at depth in Baffin Bay. Between 21–43% of surface CRAM is removed at depth. These results suggest both CRAM and carbohydrates are major LDOM constituents – contradicting the existing CRAM cycling paradigm and further constraining the long-term persistence of deep ocean DOM.

## Abstract (web summary)

A study using total dissolved organic matter (DOM) proton nuclear magnetic resonance spectroscopy found rapid removal of surface-derived carbohydrates and carboxyl-rich alicyclic molecules at depth, challenging our notions of deep ocean DOM storage.

## Introduction

Understanding the nature and cycling of dissolved organic matter (DOM) is of paramount importance for constraining the marine carbon cycle. Given the size and great age (4000–6000 years) of the DOM reservoir^1^, DOM acts as a capacitor for deep ocean carbon storage, mitigating climate on timescales of centuries to millennia. DOM is likely comprised of millions of different compounds^2^ yet the majority of DOM remains uncharacterized at the molecular level^3^, or elusive to current direct isolation techniques. This has precluded our ability to quantify DOM molecular transformations and cycling.

The Arctic Ocean is warming four times faster than the rest of the planet^4^. Resulting ecosystem level impacts on phytoplankton communities and blooms, together with changes in DOM production, are likely to alter the microbial loop and sequestration of DOM in these regions^5^. Baffin Bay is a known region of significant labile DOM (LDOM) production^6^. However, little is known about recalcitrant DOM (RDOM) cycling in this region and the biogeochemical cycling of DOM within Baffin Bay remains poorly constrained.

Detailed chemical analysis, and in particular NMR measurements, have shaped our understanding of DOM composition and cycling. Early studies using tangential-flow ultrafiltration (UF) for the first isolation of high molecular weight (HMW; >1 nm) DOM allowed for both isotopic and chemical characterization of DOM. Through ^13^C-NMR measurements it was estimated that ~50% of surface ultrafiltered high molecular weight DOM (HMW DOM; >1000 Da) was carbohydrates, whereas both surface and deep HMW DOM were composed of primarily aliphatic molecules and carboxylic acids^7,8^. It was inferred that surface carbohydrates represented a major carbon rich substrate for biological respiration in the upper water column which could then be advected and slowly degraded to subsurface waters.

Subsequent ^1^H-NMR analyses revealed neutral sugars, acetate, and lipids shared similar depth trends, suggesting the biosynthetic surface production and rapid removal of complex macromolecular heteropolysaccharides (HPS; including acyl oligosaccharides, and N-acetyl amino polysaccharides)^9,10^. This was later confirmed through mass-spectrometry studies identifying methylated hexoses–, pentoses–, 6-deoxysugars–, heptoses–, 3,6-dideoxysugars, and 1,6 anhydrosugars as major components of HMW DOM HPS^11^. This work is supported by direct measurements of total carbohydrates (TCHO) and dissolved combined neutral sugars from the Atlantic and Pacific, suggesting this to be a major LDOM component, cycling on seasonal timescales^12^. While our understanding of bioavailable or LDOM cycling through its biomolecular proxies (e.g., amino acids, sugars, fatty acids, etc.) are well constrained, little is known about the composition and cycling of refractory DOM on centennial to millennial timescales.

Early NMR studies revealed the refractory nature of marine “humic” substances within the DOM reservoir and in the deep ocean. Deep ocean HMW DOM has high abundances of carboxyl and aliphatic carbon held in highly branched and interlinked (e.g., cyclic) structures^13^. Based off ^13^C-NMR, this material is found throughout the water column, but in higher relative total abundance in deep HMW DOM, suggesting a refractory “humic” background DOM component that lasts several oceanic mixing cycles^8,14^. Using a combination of NMR and ultrahigh resolution mass spectrometry, this refractory material was later coined “carboxyl-rich alicyclic molecules” (CRAM) and was estimated to comprise ~8% of HMW DOM^15^.

Several molecular compound classes have been identified as key components of the long-lived recalcitrant RDOM pool, including dissolved black carbon, carotenoids, humic-like fluorescent DOM and deaminated peptides^16–19^. However, CRAM is the compound class most commonly referenced as a suitable proxy for RDOM formed via the microbial carbon pump^15,20,21^. Recent NMR and isotopic studies implemented a combined ultrafiltration and solid phase extraction approach to isolate HMW and low molecular weight solid phase extracted DOM (LMW SPE DOM; <1000 Da) fractions from two oligotrophic subtropical stations in the Pacific and Atlantic (ALOHA and BATS) suggest the composition of LMW SPE-DOM closely matches that of CRAM and that LMW SPE-DOM composition is invariant with depth and between the two ocean basins^22–24^. Recent long-term microbial incubations combining NMR and ultra-high resolution mass spectrometry of SPE-DOM from the south China sea found invariant DOM compositions over 180-day incubations at all depths^21^. Hertkorn and co-workers more recently estimated that CRAM comprises between 51–56% of solid phase extractable (SPE) DOM^25^.

Together, these studies highlight SPE-DOM as a suitable proxy for RDOM, one that is rich in CRAM. However, several key problems impede our ability to quantify RDOM cycling. First, the centennial timescales in which RDOM is remineralized via microbial heterotrophy and/or photochemical oxidation^26^ complicate direct observations of its biogeochemical cycling. Second, all NMR and ultra-high resolution mass spectrometer studies have relied on size– and/or chemical–fractionation methods, which can obtain at most 40–60% of DOM. To the best of our knowledge, no %CRAM estimates (as % dissolved organic carbon; DOC) within the total DOM pool have been reported. Third, NMR has only been used to estimate relative percent abundance of CRAM, precluding precise carbon budgets or estimates of “standing stock” concentrations (e.g., µmol C kg^−1^) of key RDOM components.

**Fig. 1:**
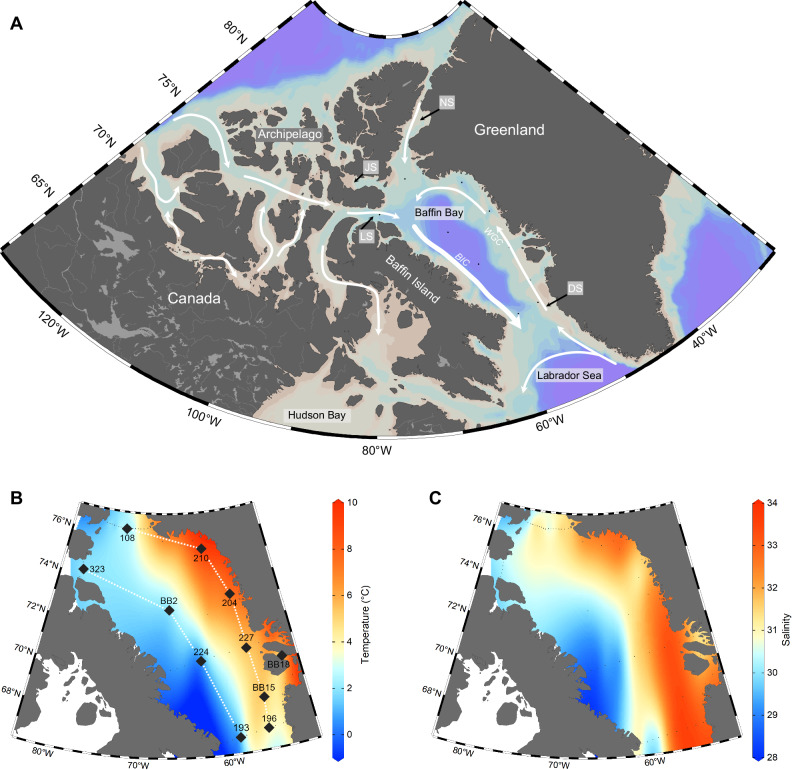
Map of major current systems (Baffin Island Current and Western Greenland Current) and sample site locations in Baffin Bay. **A** Major current systems in Baffin Bay as well as inflow and outflow currents from the Canadian Arctic Archipelago and the Labrador Sea are shown by the white arrows. The locations of major Baffin Bay gateways are denoted by small black arrows and gray boxes. These are Nares Strait (NS), Jones Sound (JS), Lancaster Sound (LS) and Davis Strait (DS). **B** Temperature data for Baffin Bay surface water is shown by the red and blue color map; stations are represented by the labeled black diamond,s and East and West transects indicated by white dashed lines. **C** Salinity data for Baffin Bay surface water is shown by the red and blue color map. This figure was adapted from ref. ^30^ in accordance with open access Creative Commons Attribution License (CCBY) license terms. Minor changes were made to the currents represented in (**A**) for improved accuracy.

Here we present total DOM ^1^H-NMR data from 10 stations in Baffin Bay (Fig. 1) using a water suppression method^27,28^. We use total ^1^H-NMR abundance data together with DOC concentrations and known functional group H:C ratios to quantify the molar abundance and examine the cycling of two DOM endmembers: total TCHO and CRAM. We evaluate the utility of relative percent abundance estimates and compound concentrations (in µmol C kg^−1^) as representatives of LDOM or RDOM throughout Baffin Bay.

## Results and discussion

**Fig. 2:**
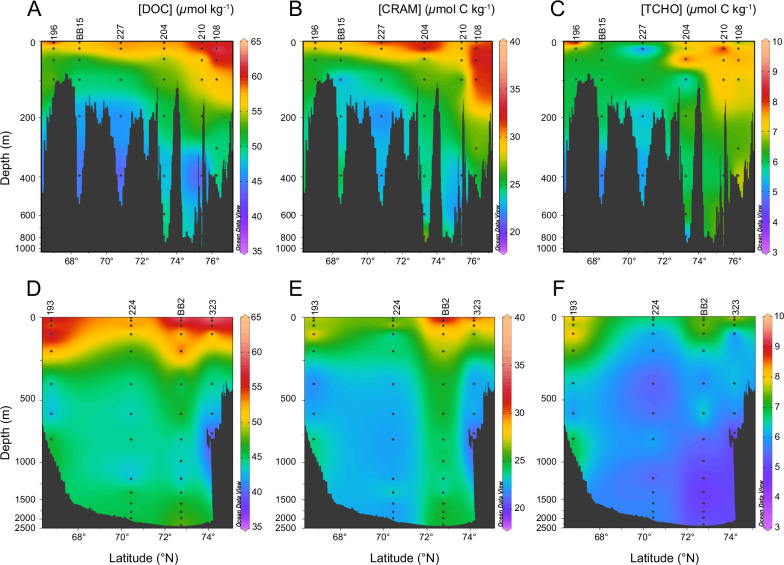
Section plots following the transects within two major current systems in Baffin Bay; West Greenland Current (A, B, C) and Baffin Island Current (D, E, F). The color map indicates the concentration of each of the compound classes; Dissolved organic carbon (DOC; **A**, **D**), Carboxyl-rich alicyclic molecules (CRAM; **B**, **E**), or total carbohydrates (TCHO; **C**, **F**) in µmol C kg^−1^. The black circles show sample locations at each station named on the top x-axis. Black regions represent the bathymetry of the seafloor. Data are summarized in Supplemental Data 1 and source data are provided as a Source Data file.

Baffin Bay is a region of high seasonal primary productivity, with a host of diverse water masses, nutrient sources^29^ and long deep-water residence times (360–690 years)^30^. For a detailed hydrographic description, see the Supplementary Information and Supplemental Data 1. Thus, changes in DOM composition, and specifically the distribution and differential cycling of LDOM vs. RDOM components (e.g., TCHO and CRAM) within Baffin Bay is expected. DOC concentrations (Fig. 2A, D) ranged from 36.6 to 64.8 µmol kg^-1^ with higher average concentrations in the surface 100 m (55.2 ± 5.4 µmol kg^−1^; *n* = 43) vs. at depths over 200 m (45.5 ± 3.5 µmol kg^−1^; *n* = 36). Total ^1^H-NMR integrations of TCHO regions and CRAM (see methods) indicate TCHO comprises between 7−19% and CRAM comprises between 37–59% of DOM in Baffin Bay (Fig. 2). Our %TCHO estimates are on the same order of magnitude as mean %TCHO yields previously reported for North Atlantic water masses using the spectrophotometric MBTH method (3-methyl-2-benzothiazoline hydrazone; a colourimetry measurement of sugar aldehydes; 12–20%)^12,31^. Based on ^1^H-NMR integrations (see methods), we observe several specific DOM biomarkers are driving composition variability within Baffin Bay (e.g., CH_3_-deoxysugars, protein/peptides, methanethiol, and N-acetyl amino sugars).

Our total seawater %CRAM estimates are both surprising and unexpected. Whereas the aforementioned UF and SPE studies estimated CRAM to comprise 8–28% of the DOM pool^15,25^, our results suggest that CRAM within total DOM is up to 7 times more abundant. For a detailed comparison of SPE vs. total seawater DOM ^1^H-NMR, see the Supplementary Discussion (Fig. S1; Table S1). These data suggest a significant fraction of total CRAM is not retained by either UF or SPE methods. This “missing” CRAM has also eluded detection by traditional isolation and measurement (e.g., high-resolution mass spectrometry) techniques. Hertkorn et al.^25^ found that 51–56% of SPE-DOM was CRAM. If we assume 40–61% as a nominal SPE-DOC recovery^32,33^, SPE-DOM CRAM would comprise between 20–34% of total DOC. HMW DOM CRAM was found to contain 8% of total DOC^15^. Thus, the fraction of CRAM that can be quantified using traditional isolation techniques comprises between 28–42% of the total DOC pool. Our total DOM ^1^H-NMR results suggest CRAM comprises between 37–61% of total DOC, suggesting a significant fraction of total CRAM (up to 31%) has evaded detection by coupling these earlier techniques. This is consistent with recent work showing that SPE has a lower affinity for isolating marine humic substances due to an inherent SPE-DOM molecular composition bias^34^. If similarly high %CRAM is observed in the Atlantic and Pacific Oceans, our results would suggest CRAM is the most abundant, identifiable DOM compound-class.

Percent carbon yield estimates are commonly used to evaluate broadscale changes in DOM composition, and thus to infer differential cycling of a compound-class relative to the total DOM pool^7,15,35^. Despite some station and depth specific variance, and wide overall ranges in, %TCHO and %CRAM (Figs. S2 and S3), we do not observe statistically significant differences between average %yields in the surface (<100 m; %TCHO = 13 ± 2% and %CRAM = 50 ± 4%; *n* = 43) and deep waters (>200 m: %TCHO = 13 ± 2% and %CRAM = 51 ± 4%; *n* = 36). A one-way ANOVA test comparing surface versus deep % abundance values confirms the similarity of surface versus deep %TCHO (F(1, 87) = 0.11, *p* = 0.74) and %CRAM [F(1, 18) = 0.0004, *p* = 0.98] values. Similarly, a one-way ANOVA resulted in no significant average difference between the two transects (Baffin Island Current vs. Western Greenland Current) for surface %TCHO (F(1, 38) = 2.87, *p* = 0.10), deep %TCHO (F(1, 41) = 0.98, *p* = 0.33), surface %CRAM (F(1, 38) = 0.31, *p* = 0.58) or deep %CRAM (F(1, 41) = 1.93, *p* = 0.17). Heterogeneous %TCHO distributions at depth are difficult to explain but could represent fresh carbohydrate contributions via hydrolysis of sinking particles, resuspension from local shelf/slope regions or recent water mass ventilation. We note that similarly homogeneous %TCHO depth profiles were observed in North Atlantic waters above 40°N (%TCHO = 18–25%) together with relatively uniform DOC concentrations^12^. Our %TCHO estimates do not distinguish between poly- and monosaccharides, and thus do not preclude the microbial transformation of surface polysaccharides to more degraded monosaccharides which persist at depth as has been previously inferred^7^. The lack of clear broad scale trends in %TCHO and %CRAM suggests these proxies for DOM compositional change are perhaps less useful than compound-class carbon concentrations (e.g., µmol C kg^−1^) for understanding DOM biogeochemical cycling in this region.

In contrast to our %yield data, average CRAM and TCHO concentrations clearly decrease with depth from 26.9 ± 1.9 µmol C kg^−1^ and 6.9 ± 0.6 µmol C kg^−1^ above 200 m (*n* = 43) respectively, to 23.9 ± 1.5 µmol C kg^−1^ and 5.9 ± 0.7 µmol C kg^−1^ below 200 m (*n* = 43) (Fig. 2). A one-way ANOVA test comparing the surface versus deep concentration values confirms the statistically significant difference of the surface versus deep TCHO (F(1, 78) = 25.29, *p* < 0.001) and CRAM (F(1, 78) = 36.46, *p* < 0.001). Carbohydrate concentrations estimated via our ^1^H-NMR approach are similar to those measured directly using the MBTH method^7,12^, and contain between 50-70% more TCHO than SPE-DOM (Table S1), suggesting the technique more closely approximates seawater TCHO concentrations vs. traditional D_2_O NMR experiments. On average, we observe a 1.0 µmol C kg^−1^ loss of TCHO with depth, and a persistence of 6.0–7.6 µmol C kg^−1^ recalcitrant TCHO in Baffin Bay Deep Water. Previous work in the North Atlantic using the MBTH method observed a 3.0 µmol C kg^−1^ loss of labile TCHO with depth and the persistence of 5–10 µmol C kg^−1^ recalcitrant TCHO at depth which was dominated by both hydrolysis-resistant polysaccharides (e.g., chitin and cellulose) and mono-saccharides^31^. To first order, we observe a similar amount of labile vs. recalcitrant TCHO within the upper water column and deep waters of Baffin Bay, respectively.

We observe qualitative trends in surface (0–100 m) [CRAM] and [TCHO] abundance in our transects (Fig. 2). For example, within the Western Greenland Current (WGC) [CRAM] and [TCHO] values appear to increase from South (Station 196: ~25–30 µmol C kg^−1^ and 6–7 µmol C kg^−^^1^, respectively) to North (Station 108: ~30–40 µmol C kg^−1^ and 6–8 µmol C kg^−1^, respectively) with water mass advection of the current along the Greenland shelf. Similarly, [CRAM] and [TCHO] values decrease slightly along the Baffin Island Current (BIC) extension from North (Station 323: ~28–34 µmol C kg^−^^1^ and 7–8.5 µmol C kg^−^^1^, respectively) to Central Baffin Bay (Station 224: ~24–30 µmol C kg^−^^1^ and 6–7.5 µmol C kg^−^^1^, respectively). Along the Greenland transect, we also observe increasing [CRAM] and [TCHO] within West Greenland Irminger Water (WGIW) and Transitional Mode Water (TrW)^36^ between 100–400 m (Fig. 2). These observations would be consistent with CRAM and TCHO production within the highly productive North Water Polonya (Station 108)^37,38^ but also with past CDOM observations of “humification” within WGIW and apparent oxygen utilization^39^ and the southerly removal and dilution of terrestrial DOM, as evidenced from a CDOM anomaly (Fig. S4) exported from the CAA within the BIC extension. We note when comparing average surface water values of [CRAM] and [TCHO] from stations within both transects, these observed differences fall outside statistical significance given surface variance in observed [CRAM] (±3–4 µmol C kg^−^^1^) and [TCHO] (±1–2 µmol C kg^−^^1^) values in the upper 100 m. Future work should focus on more detailed evaluations of DOM transformation such that changes can be quantified within these two disparate current systems.

We also observe significant decreases in CRAM (3.0 µmol C kg^−^^1^) with depth in Baffin Bay—from 26.9 µmol C kg^−^^1^ in the upper 100 m (*n* = 43) to 23.9 µmol C kg^−^^1^ below 200 m (*n* = 36). Decreasing CRAM concentrations with depth generally opposes the current paradigm of marine CRAM cycling. CRAM is often inferred to have an invariant, biologically refractory chemical composition that accumulates and persists in the deep ocean as the result of slow heterotrophic microbial activity over timescales of ocean mixing^21–24^. While our results generally support the idea that a large reservoir of CRAM (average = 23.9 µmol C kg^−^^1^; *n* = 36) persists in deep Baffin Bay, an average 3.0 µmol C kg^−^^1^ loss of surface CRAM suggests that a significant fraction of the total CRAM pool (~11% of total DOC) is produced in the surface ocean. We hypothesize the most like mechanisms of CRAM production to be autotrophic, through microbial heterotrophic degradation of photosynthate C, or via photochemical oxidation of similar compound classes (e.g., sterols or carotenoids)^17^. It is likely labile CRAM is both biologically and photochemically available, allowing it to be rapidly removed in the surface ocean. A partial least squares linear regression of CRAM (x) vs. TCHO (y) values in the BIC extension (*σ*_θ_ = 26.0–27.0 kg m^-3^, Stations 323, BB2, 224, 193; 20–100 m) resulted in a weak positive correlation (R^2^ = 0.36, DF = 8, *p* = 0.07), supporting the interpretation that labile CRAM and TCHO cycle similarly within the same water mass. Subtracting minimum CRAM concentrations below 200 m from maximum CRAM concentrations in the upper 100 m for each station sampled, we estimate that labile CRAM removal could be much higher (e.g., between 5.0–15.1 µmol C kg^−^^1^; 17–42%). Loss of labile surface CRAM with depth is consistent with recent studies observing some bacterioplankton-specific oxidation of model CRAM proxy compounds (e.g., deoxycholate)^40^ and the selective microbial degradation of CRAM produced through the incubation of testosterone induced metabolites^41^. Our observations suggest rapid removal of surface CRAM is three times higher than that for TCHO. However, these changes do not directly support “priming” (i.e., additional removal of RDOM in the presence of LDOM) since CRAM-depth gradients are not correlated to higher surface [TCHO] values. Together with previous studies, our data suggest that CRAM cannot simply be considered RDOM, but instead that a “two-pool model” of labile vs. recalcitrant CRAM cycling exists in this region. While further work is needed to confirm the differential cycling of individual CRAM compounds, we hypothesize that CRAM contains a continuum of molecular forms with differing chemical composition (isomers or heteroatom abundance), ^14^C-ages, molecular sizes, and biological reactivities. Similarities in the distributions of total DOC to CRAM concentrations (given by the relatively consistent %CRAM we observe), suggest that a significant fraction of the DOM cycle in Baffin Bay is mediated by the production and removal of CRAM.

Using our CRAM concentrations together with recent hypsometry and volumetric estimates of Baffin Bay, we calculate the mass of CRAM and TCHO carbon stored within deep Baffin Bay. Jakobsson and co-workers estimated surface (0–200 m) and deep (200–2450 m) Baffin Bay to contain 6.67 × 10^4^ and 3.69 × 10^5^ km^3^ volume, respectively^42^. Using average CRAM and TCHO concentrations of 23.9 µmol C kg^−^^1^ and 5.7 µmol C kg^−^^1^ within deep Baffin Bay we estimate that 106.2 Tg C of CRAM and 25.5 Tg C of TCHO are stored in deep Baffin Bay. Using previously established ^14^C residence times, recalcitrant forms of deep CRAM and TCHO likely persist for centuries (360–690 years)^30^. Similarly, using average surface CRAM and TCHO concentrations (<200 m) of 26.9 µmol C kg^−^^1^ and 6.8 µmol C kg^−^^1^, we estimate 21.6 Tg C of CRAM and 5.5 Tg C of TCHO in the surface of Baffin Bay. Subtracting the difference of surface vs. deep CRAM and TCHO concentrations would thus imply in the semi-annual removal of 2.4 Tg C and 0.8 Tg C of biologically labile CRAM and TCHO molecular populations.

Taken together, our total seawater ^1^H-NMR results are consistent with recent work challenging the notion that CRAM and TCHO can be simply considered canonical proxies for RDOM and LDOM, at least for the Baffin Bay region. We report the highest total DOC CRAM concentrations to date – suggesting that, as a compound-class, CRAM (37–59%) comprises the largest identifiable fraction of the DOM pool that has evaded other methods of detection due to size- and chemical-fractionation biases (e.g., ultrafiltration and SPE techniques). Surface to deep gradients in CRAM occur largely in concert with changes in DOC concentrations. We hypothesize a large portion of the marine DOM cycle is mediated through both autotrophic production and heterotrophic cycling of this population of chemically-distinct molecules. While more work is needed to constrain CRAM distributions and cycling in the global ocean, our study suggests that up to 21–43% of surface CRAM is semi-labile material, at least within high-productivity Arctic ecosystems. These observations change our current understanding of the DOM cycle in regions of rapid Arctic climate and ecosystem change. While significant loss of bioavailable CRAM and TCHO is observed, we also hypothesize the long residence time of Baffin Bay Deep Water (360–690 years)^30^ will result in long-term storage of recalcitrant CRAM and TCHO in deep Baffin Bay. If true, we hypothesize that deep CRAM and TCHO populations should have greater average ^14^C-ages, and potentially molecular diversity, resulting from slow microbial heterotrophic degradation imparting recalcitrance before entering meridional overturning circulation in the North Atlantic. This hypothesis will be readily testable with future development of CRAM isolation techniques. Future temporal studies focused on assessing changes in CRAM and TCHO will be paramount in constraining Arctic DOM carbon budgets, and assessing how the Arctic DOM reservoir might respond in the face of anthropogenic climate change.

## Methods

### Sample collection

Seawater samples were collected aboard the CCGS Amundsen on Leg 2a of the Arctic Net expedition in Baffin Bay between July 5^th^ to 25^th^, 2019 (Fig. 1). At sea, samples collected from depths shallower than 400 m were filtered using a custom-built 306 Stainless Steel 70 mm filter manifold. The manifold was pre-cleaned with 10% HCl and copious amounts of Milli-Q water (3 ppb TOC) and dried prior to use. The manifold was loaded with pre-combusted (540 °C, 2 h) 70 mm glass fiber filters in two layers. A 2.0 µm quartz filter (Whatman QMA) was placed on top of a 0.7 µm borosilicate filter (Whatman GF/F). Dissolved organic carbon (DOC) and ^1^H-NMR samples were collected by filtering water from 11 L Niskin-type Bullister bottles via acid-cleaned PTFE and silicon tubing into pre-combusted, 10 mL pre-scored borosilicate ampules (Wheaton part#176780) for ^1^H-NMR, and into pre-cleaned (overnight 10% HCl soak) 60 mL high density polyethylene (HDPE) bottles (Fisher Scientific, #03-331-32B) for DOC. HDPE bottles were immediately frozen and stored at −20 °C for future analysis. Ampules for ^1^H-NMR samples were overfilled with 5x sample volume to reduce potential contamination. After collection, ampules were capped with pre-cleaned polypropylene ¼” column caps until they could be poisoned with 1 drop (~50 µL) saturated mercuric chloride (HgCl_2_, Fisher #M1136-100G), and flame sealed with a small butane torch. To further minimize contamination, a separate dram vial of saturated HgCl_2_ was used for each station. Poisoned and flame-sealed samples were cooled, homogenized, checked for leaks, and stored in the dark at room temperature until further sample processing at the University of Ottawa.

### Dissolved organic carbon concentrations

A total of 98 seawater samples were prepared for DOM analysis at the University of Ottawa. Briefly, samples were rapidly thawed, homogenized and transferred into pre-combusted (540 °C/2 h) 20 mL glass autosampler vials (Fisher Scientific, #03-375-25) and acidified to pH < 2 with 50 µL of 12 M HCl and capped with pre-cleaned (10% HCl) PTFE lined silicone septa caps. Samples were re-frozen, packaged, and shipped to the CERC.OCEAN laboratory (Dalhousie University) for DOC concentration analysis using a Shimadzu TOC-L Total Organic Carbon (TOC) analyzer. A Five Point Calibration was performed prior to sample analysis to create a TOC calibration curve using reagent grade KHP (potassium hydrogen phthalate) in Milli-Q water. A Milli-Q Water blank and 100 μL of DOC Consensus Reference Material (CRM; Batch 20 Hansell Lab) was injected prior to the analysis of our samples and was repeated after every six sample analyses for quality control. Samples were injected at least three times, with a maximum of five injections, until a CV better than ±2% was achieved.

### Solid phase extraction (SPE)

SPE extraction was adapted from ref. ^32^ with slight modifications. Agilent Bond Elute PPL 1 gram 6 ml cartridges were conditioned with 3 cartridge volumes of Optima (LC-MS) grade methanol, followed by washing with 1 cartridge volume of Milli-Q water. An 800 ml acidified water sample (pH 2 with 12 M trace metal HCl) was then passed through the PPL-SPE cartridge under vacuum assistance (5 psi). The samples were washed with 6 ml of pH 2 Milli-Q water. The cartridges were allowed to dry completely before elution. The extract was eluted with 6 ml of Optima (LC-MS) grade methanol.

Care was taken to account for the SPE resin blank and to fully remove residual methanol in SPE-DOM samples. The methanol-extracted samples were dried using a Labconco Centrivap (chamber at 35 °C and cold trap at −85°C). The SPE-DOM was then reconstituted in 1 ml of Milli-Q water, dried again with the Labconco Centrivap to minimize DOC measurements being affected by residual methanol. This re-dissolution in 1 ml of Milli-Q and drying was repeated two more times (3 times in total). The dried samples were then shipped to the University of Ottawa for NMR analysis. Analysis of methanol blanks and samples by NMR suggested no presence of residual methanol, and minimal SPE resin contributions to our SPE-DOM spectra (e.g., <0.0 µM C kg^−1^). SPE extraction yields ranged from 39–55% of total DOC (Table S1).

### Proton (^1^H) NMR sample preparation

Due to the sensitivity of the water suppression ^1^H-NMR analysis technique and extremely low concentrations of seawater DOM in our samples (40–80 µmol kg^−1^), rigorous cleaning of NMR tubes and meticulous sample preparation are required. Also, since NMR tubes will bend if baked in an oven and damage the probe, these tubes cannot be pre-combusted prior to analysis. Instead, our NMR tubes are cleaned with Milli-Q water (TOC = 3ppb) and the sample itself. To minimize residual particulate organic matter (POM) contamination that interferes with the water suppression ^1^H-NMR method, ampules were centrifuged at a relative centrifugal force (RCF) of 1900 × *g* for 20 min and allowed to come to a rest in the centrifuge, gradually, without braking. This effectively eliminated POM contamination in the samples. After centrifugation, the sample ampule was gently cracked open, and sample was gently pipetted from the top using a new baked (540 °C/2 h) 2 mL pipet into NMR tubes (Wilmad Precision 500 MHz; part #665000575). The aspiration was done very carefully so as not to resuspend any residual POM in the sample.

Prior to sample loading, NMR tubes were rinsed five times with ~1 mL of 18.2 MΩ (Milli-Q) water (TOC = 3ppb) using a pre-baked (540 °C, 2 h) 2 mL Pasteur pipet. For each rinse, ~1 mL of Milli-Q water was added, the NMR tube capped and shaken vigorously to ensure the tube was fully rinsed, then decanted into a waste container. The Milli-Q water used for rinsing NMR tubes was stored in a pre-baked (540 °C, 2 h) 1000 mL amber Boston round bottle with an acid cleaned (10%HCl) PTFE lined cap. The same Milli-Q water was used for rinsing all NMR tubes used in this study. After the Milli-Q water rinse, the NMR tube was then rinsed five times with ~0.5 mL of sample seawater dispensed by a separate pre-baked (540 °C/2 h) 2 mL pipet. Prior to sample loading, a new baked 2 mL pipet was used to dispense one drop ( ~ 50 μL) of deuterium oxide (Aldrich 99.9% D_2_O, part#151882−10 × 0.6 ML) into the NMR tube. Next 600 µL of sample seawater was added to the NMR tube containing the one drop (50 µL) of D_2_O to a height of 4.00 ± 0.05 cm from the bottom of the NMR tube. Maintaining this sample meniscus height is important for the water suppression method to work well. Finally, the NMR tube was flame sealed, cooled, and homogenized. The remaining sample was transferred to a new baked 10 mL ampule and flame sealed as an archive in case a repeat measurement was needed. The NMR tube was stored at room temperature and in the dark prior to NMR analysis. SPE-DOM samples were prepared in an identical method to that above for water suppression NMR analysis. For traditional D_2_O ^1^H-NMR analysis, SPE samples were deuterated by drying to completeness with centrifugal evaporation (−85 °C), re-suspending in 99.9% D_2_O, sonicating for 20 min to fully exchange H/D and this process repeated three additional times prior to loading into NMR tubes for immediate analysis.

### ^1^H-NMR measurement and processing

Samples were analyzed on the Bruker Avance IIIHD 600 MHz NMR Spectrometer with a Bruker 5 mm inverse H-C/N-D Cryoprobe at the University of Ottawa following the methods set forth by Lam and Simpson^28^. A watergate W5 water suppression pulse sequence (zggpw5) with gradients and a double echo was used. The ^1^H 90° pulse was calibrated for each sample, and generally had a pulse length of 19 μs. The shaped 180° pulse was 2 ms in length, and the gradient pulse length was 1 ms in length. A total of 13,528 transients using 4 dummy scans were acquired, with an acquisition time of 2 s, a pre-scan delay of 30 μs, a recycle delay of 0.01 s, a gradient recovery delay of 200 μs, a binomial water suppression delay of 120 μs, and a loop count of 1000. Each total seawater sample experiment had a total duration of 15 h and 15 min. SPE-DOM watergate experiments were run under identical conditions, but only for a total of 1024 transients (1 h). The ^1^H experiments in D_2_O without solvent suppression (See Supplementary Information) consisted of a single 30° pulse of ~2.76 μs in length on ^1^H followed by a 4 s acquisition time using a 0.01 s recycle delay and acquiring 960 transients with no dummy scans (1 h).

NMR spectra were processed via Fourier transform using TopSpin v. 4.0.7 with zero filling using manual phase correction and initially the automatic baseline correction. Data was imported into Microsoft Excel for normalization. ^1^H NMR chemical shifts were referenced to (0.0) ppm using the ^1^H signal of tetramethyl silane (TMS = 0.2) ppm. The spectra were phased to ensure that the regions on either side of the water peak (4.8 ppm) and on either side of the TMS peak (0 ppm) were approximately flat by setting PHC0 = 159.837 and PHC1 = −126.735—determined experimentally to result in correct phasing on the spectrometer at uOttawa for this technique. A manual polynomial baseline-correction was applied to ensure spectra had equal baseline intensity for the TMS and water peak regions.

Phased and baseline corrected data was imported into Microsoft Excel for normalization and integration. Data were “smoothed” by taking the average of every *n* = 10 data points from the available 65,536 data points in each raw spectra (from 15 to −5 ppm). Only data between 5.0 ppm and 0.2 ppm was used in our data analysis, resulting in 1430 data bins. The averaged data of each spectrum was area normalized by dividing each intensity data bin by the sum of all the intensities in the sample spectrum.

### Spectral integration of compound classes and statistical analysis

^1^H-NMR is quantitative (i.e., the relative intensity of the chemical shift spectral regions indicates the number of protons of a given functional group), and thus integration of spectral areas can be used to determine the percent relative abundance of functional groups or “compound classes” (e.g., CRAM, CHO-carbohydrates, etc). The chemical shift (δH) ppm ranges of key compound classes were identified in sample spectra using after performing a 2D correlation analysis (in 2DShige software) and a principal component analysis (Matlab v. 9.9.0.1538559) and are summarized in Table S2 following Fox and co-workers^27^. Our integration and prescription of H:C ratios for each compound class are further clarified in the Supplementary Information text, Table S2 and Fig. S5. Area normalized spectra were integrated via trapezoidal elements in Microsoft Excel using Eq. 1.

1 $${T}_{n}=\frac{\left({I}_{n+1}+{I}_{n}\right)*\left({P}_{n}-{P}_{n+1}\right)}{2}$$

Where *T*_*n*_ is the trapezoidal element of data point *n*, *I*_*n*_ is the intensity of data point *n*, and *P*_*n*_ is the ppm value of data point *n*. The sum of all the intensities for a specific sample was then calculated (*S*_*tot*_), as well as the sum of all the intensities of a specific compound class for that sample (*S*_*x*_). The percent relative abundance (*A*_*x*_) was then calculated using Eq. 2.

2 $${A}_{x}=\frac{{S}_{x}}{{S}_{{tot}}}*100\%$$

The percent relative abundance (%H) of each compound class was next divided by the H:C ratio^43^ (*H:C*_*x*_) for that compound class (Table S2) to find the percent abundance of carbon (*C*_*abundance*_ = %C). The abundance of carbon was calculated using Eq. 3.

3 $${C}_{{abundance}}=\frac{{A}_{x}}{{H:C}_{x}}$$

*C*_*abundance*_ was then multiplied by measured sample dissolved organic carbon concentrations ([DOC]) to determine compound class carbon-based concentration [Compound] in µmol C kg^−1^ using Eq. 4.

4 $$\left[{Compound}\right]={C}_{{abundance}}*[{DOC}]$$

Five sample duplicates, taken from a second ampule collected from the same Niskin bottle at sea, were measured to assess the total analytical reproducibility of CRAM and TCHO relative % abundance and carbon concentration. Duplicate samples were prepared and measured by NMR analysis weeks to months after the original was run. Analysis of replicates data can be found in Table S3. Sample replicates resulted in %CRAM and %TCHO standard deviation ranges of ±0.2–2.6% and ±0.5–0.6%, respectively. [CRAM] and [TCHO] concentration standard deviation ranges were ±0.1–1.6 µmol kg^−1^ and ±0.3–0.4%, respectively.

Ocean Data View (v. 5.5.1) software was used to create section plots (Figs. 2 and S1) following the two major current systems in Baffin Bay (West Greenland Current and Baffin Island Current). The color maps depict how the concentration and abundance of DOC, CRAM, and TCHO change with depth and latitude.

## Supplementary information

Supplementary Information

Description of Additional Supplementary Files

Supplementary Data 1

## Source data

Source Data

Peer Review File

## Data Availability

Source data are provided with this paper. They are available as a data files (Supplemental Data 1.xlsx and Source Data.xlsx) in the Supplementary Information and can also be accessed through the Open Science Framework via 10.17605/OSF.IO/NHY2S with CC0 1.0 Universal access conditions. Reprints and permissions information is available online at www.nature.com/reprints. Source data are provided with this paper.
