# Supplementary material for: Cycling of labile and recalcitrant carboxyl-rich alicyclic molecules and carbohydrates in Baffin Bay
Source: Nat Commun. 2024 Oct 9;15:8735. doi: 10.1038/s41467-024-53132-5 (PMC11464691; doi:10.1038/s41467-024-53132-5)
Supplement: Supplementary file 1 — Supplementary Information [file 41467_2024_53132_MOESM1_ESM.pdf]

Supplementary Information:

Cycling of labile and recalcitrant carboxyl-rich alicyclic molecules and carbohydrates in Baffin Bay

Kayla McKee[1], Hussain Abdulla[2], Lauren O'Reilly[1], Brett D. Walker[1,3*]
[1]Department of Earth and Environmental Sciences, University of Ottawa, Ottawa, ON, Canada
[2]Department of Physical and Environmental Science, Texas A&M University-Corpus Christi, Corpus Christi, TX, United States
[3]Department of Earth System Science, University of California, Irvine, CA, United States

[*]Corresponding Author: brett.walker@uci.edu

Supplementary Discussion:

Comparison of total seawater vs. SPE-DOM using water suppression and traditional ^{1}H-NMR:

The water suppression method slightly attenuates the carbohydrate region between 4.5-5.5ppm, which could lead to an underestimation of the carbohydrate contribution, as described by Lam et al. (2008) and Fox et al. (2018). However, we believe this effect will be minimal for several reasons. First, the suppression effect will be consistent across all samples, which is crucial for a comparative study like ours. Second, the carbohydrate anomeric proton is not the major hydrogen bond of the carbohydrate, representing less than 20% of the total carbohydrate H-NMR region. Third, we still quantify the other major ^{1}H-NMR bands of carbohydrates, and by using the correct H-ratio, we can quantify the majority of carbohydrates. And finally, the effect of water suppression on CRAM (Carboxyl-Rich Alicyclic Molecules) will be negligible since the CRAM region was integrated from 0.2-3.2 ppm, which is lower than the water suppression region of 4.5-5.5 ppm.

To verify the impact of water suppression on the carbohydrate region, but also to explore the differences in DOM composition between total seawater and more common DOM isolates, we have performed a direct comparison of contemporaneous total seawater DOM vs. Solid Phase Extracted (SPE) PPL DOM for n = 4 Baffin Bay and the Canadian Arctic Archipelago samples from various depths. We have also performed n = 2 traditional D_2O ^{1}H-NMR experiments on SPE-DOM to compare our NMR methods to those more commonly used in the literature. Methods for these extractions and NMR analysis are provided in the main text Methods. The results of this comparison are summarized in Table S3 and Figure S4.

A comparison of intensity normalized spectra for all three treatments (watergate seawater, watergate PPL, PPL D_2O), reveals a few key spectral differences. First, total seawater had more spectral area than watergate PPL and PPL D_2O experiments (Figure S4). Surprisingly, watergate PPL and PPL D_2O experiments had smaller integrated TCHO and in some cases aliphatic (0-0.5ppm) regions than for total seawater. By integrated area, total seawater samples contained 59±2 %CRAM and 13.9±1.1 %TCHO, whereas watergate PPL and PPL D_2O contained slightly less – 52.1±1.5% and 52.5±0.4 %CRAM, and 11.6±0.9% and 9.5±0.9 %TCHO, respectively. These %CRAM estimates are consistent with previous estimates of CRAM from SPE-DOM[1]. A

comparison of [CRAM] and [TCHO] concentrations revealed more striking differences, with total seawater spectra containing between 50-70% more [CRAM] and [TCHO] vs. watergate PPL spectra. Watergate PPL and PPL D_2O spectra had nearly identical [CRAM] values (Table S3), but very different [TCHO] values, with the latter being 22-40% lower due to attenuation of this region due to residual water in the D_2O experiment. This was surprising given much care was taken to fully deuterate these SPE-DOM splits (e.g. repeatedly deuterate, sonicate, Centrivap (-85°C) to dryness). An exploration of [CRAM]:[TCHO] ratios across sample types and NMR methods suggests nearly identical ratios of CRAM and TCHO are measured in total seawater and watergate PPL samples (n=4 average of-6±9% difference). However, the attenuation of TCHO in the D_2O experiments resulted in a much higher ratio.

Together, the results suggest our methods are an overall improvement from both traditional D_2O ^1H-NMR experiments and SPE isolates. Watergate spectra appear to be more comprehensive in DOM characterization, accurate, and have much higher spectral resolution and yield higher [CRAM] and [TCHO] quantification over that of D_2O experiments. The similarity in PPL vs. total seawater [CRAM]:[TCHO] ratios suggests that SPE does not preferentially isolate CRAM over TCHO but seemingly isolates similar sub-fractions of both compound classes. Given the paucity of global SPE-DOM %CRAM data, and a known chemical preference of the PPL resin, it is plausible SPE isolates a similar CRAM population (and concentrations) throughout the water column, which would be more consistent with recent LMW-SPE DOM isotopic and NMR studies[2,3].

Baffin Bay – Hydrographic and Biogeochemical Background information:

We present the hydrographic background of Baffin Bay stemming from comments made in review. The following text was modified and adapted from our past articles (Zeidan et al., 2022)[4] and (Fox and Walker, 2022)[5]:

Baffin Bay is a large marginal sea lying between Kalaaliit Nunaat (Greenland) and Qikiqtaaluk (Greenland), connected by straits to the Arctic Ocean and the Atlantic Ocean. Baffin Bay is characterized by dynamic surface currents environment, with a stark contrast in oceanographic conditions (e.g., SST, salinity, fluorescence, and nutrients) in Western vs. Eastern Baffin Bay (Figure 1). The BIC is a southerly flowing current containing Arctic Water (AW) to

depths of 300 m and has water temperatures <-1.6°C and salinities <33.8 which exits Davis Strait into the Labrador Sea[6]. AW enters Baffin Bay via the Northern Gateways (Nares Strait and Lancaster Sound) and is modified through glacial and sea ice discharge[7]. In Eastern Baffin Bay, relatively warm and salty waters enter from the North Atlantic, forming the West Greenland Current (WGC), and flow north along the Greenland shelf. The WGC is a continuation of the East Greenland Current (EGC), composed of Arctic waters flowing southwards along the eastern coast of Greenland, with contributions from the warm and saline Irminger Current[8]. As the WGC progresses northward, it becomes fresher and cooler, due to mixing with glacial meltwater and mixing with central Baffin Bay water[9]. The WGC terminates near Smith Sound (75°N-77°N), before joining Arctic Water outflows from the CAA to form the BIC[10,11]. Together, the northwards-flowing WGC and southwards-flowing BIC form a cyclonic circulation pattern and sub-polar gyre in Baffin Bay.

Baffin Bay is a marginal sea ice zone experiencing full sea-ice coverage from December to April. Due to the relatively warm West Greenland Current, ice cover decreases earlier along the Greenland coast than on the western side of Baffin Bay[11]. By late summer, Baffin Bay is typically clear of extensive sea-ice, although icebergs are common, especially in proximity to fjords. Sources of freshwater in Baffin Bay include local precipitation, river runoff, sea ice, ice bergs and glacial meltwater, and the Arctic Ocean outflow. It is estimated Baffin Bay has a total river inflow of ~100 km^3 y^{-1} [12]. The eastern side of Baffin Bay also receives more meteoric freshwater input from melting of the Greenland ice sheet[8]. Work by Azetsu-Scott and co-workers[13] suggests that glacial melt on the Greenland Shelf comprises ~6% total freshwater and that Arctic outflow water from the CAA and Northern Gateways dominates freshwater flux in Baffin Bay (~60% in Western Davis Strait). The Arctic outflow water from the CAA includes Pacific ($31.5 < S < 33$), sea ice meltwater and meteoric (Mackenzie R. and CAA tributaries) water endmembers. A small portion of meteoric water enters Baffin Bay via Nares Strait from Russian rivers[14,15]. The difference in WGC vs. BIC currents results in differences in phytoplankton communities, sea ice conditions and rates of primary production in the region. For example, the timing and intensity of phytoplankton blooms is different on the Eastern vs. Western side of Baffin Bay and has been observed using CTD fluorescence values of the deep chlorophyll-a maximum (DCM)[5]. The depth of the DCM in our sample stations ranged from 13 to 44 metres, with an average of depth of 32±10 metres (n=11).

Annual POM flux is generally higher on the eastern side of northern Baffin Bay relative to the western side[16].

At depth, several water masses are present in Baffin Bay. West Greenland Irminger Water (WGIW) is defined by salinities >34.1, potential temperatures >2°C, and a density anomaly range of 27.3 to 28 kg m^{-3} [7]. WGIW originates from warm, saline water found in the Irminger Sea. Transitional Water (TrW) found below 250 m, is modified AW that has mixed with WGIW[7]. The TrW typically has temperatures >2°C, salinities <33.7, and σ_θ of 25.5 – 27.3 kg m^{-3} [7]. Baffin Bay Deep Water (BBDW) has a temperature and salinity of 0°C and ~34.5, respectively, and is found between 1200 and 1800m[7]. Baffin Bay Bottom Water (BBBW) has a temperature and salinity of -0.4°C and ~34.5, respectively. BBBW is more difficult to distinguish using temperature, salinity, and water mass density alone[17]. Our recent study of dissolved inorganic carbon (DIC) radiocarbon (^{14}C) ages in Baffin Bay suggests a very long residence time of BBDW (360-690 years)[4].

Only a few studies of DOM exist for Baffin Bay. Using a TDAA proxy, Shen and co-workers[18] found low DOM bioavailability in the CAA due to longer residence times and extensive microbial degradation of labile DOM into semi-labile DOM. In contrast, DOM in in Baffin Bay was found to be more bioavailable with an overall autotrophic signature. This is consistent with reported optical properties of DOM suggesting *in situ* production of humic-like components in the CAA and Baffin Bay, but also higher levels of primary production in Baffin Bay. Here, the microbial transformation of organic matter can explain 49% of humic-like DOM variation in the Canadian Arctic Archipelago's Arctic outflow[19]. The distribution, cycling and elemental stoichiometry of marine vs. terrestrial DOC and DON within Baffin Bay are the focus of a follow-up paper. There are no major rivers draining into Baffin Bay that could significantly contribute terrestrial DOC. However, a synthesis of CTD based CDOM values from our research cruise shows clearly how Mackenzie and Canadian Archipelago terrestrial DOC is transported via Lancaster Sound into the BIC and exits Davis Strait (Figure S5). In our study, we were unable to sample the center of this CDOM anomaly and based on an oxygen isotope mixing model believe that the influence of terrestrial DOC on samples reported herein to be minimal (2-8% for stations adjacent to the anomaly; O'Reilly personal communication).

On the prescription of CRAM H:C ratios and sensitivity analysis:

Stemming from reviewer comments, we provide clarifications on our prescription of at H:C ratios and sensitivity analysis on CRAM concentrations. We use an H:C ratio range of 0.8 to 1.4 based on the published FTICR-MS range for CRAM. The difference between the surface and deep ocean at each specific ratio ranged from 5.0 μmol C kg^{-1} to 2.0 μmol C kg^{-1}. The average CRAM concentration in the surface samples ranged from 31.4 to 22.5 μmol C kg^{-1} (average 26.3 μmol C kg^{-1}) and from 26.4 to 20.5 μmol C $kg^{-1}$for the deep samples (average 23.0 μmol C kg^{-1}). Using a two-sample t-test, we found that these two samples are significantly different ($p = 0.05$, t-value = 5.31). Using the range of H:C ratios, we observed a decrease of 3.3 μmol C kg^{-1}, which is close to our 3.0 μmol C kg^{-1}using a specific CRAM H:C ratio of 1.0.

We avoid adopting a range of H:C ratios in our quantification of CRAM for several reasons:

1. Using a range of H:C ratios would imply that we assign the same weight to each value within the range and assume that CRAM concentration is evenly distributed across the entire H:C range. We have no evidence to support this assumption, and given the chemistry of complex mixtures, it would not be valid.
2. Relying solely on the FTICR-MS range of H:C could introduce bias since FTICR-MS, in its current settings, is not a quantitative method. High intensity of specific mass features does not necessarily indicate higher concentrations compared to other mass features.

Instead, we chose a single H:C ratio of 1.0 for the following reasons:

1. It represents an approximate average of the proposed H:C range.
2. It reflects the common backbone structure of different CRAM isomers. According to the structure proposed by Hertkorn et al. (2006), the common backbone of the proposed CRAM structure has an H:C ratio between 1.1 and 1.0, with different isomers varying by having additional side CH_3 group or an extra double bond.
3. This approach ensures that the sum of all %C percentages, including those from other major compounds (e.g. peptides, TCHO), does not exceed 100% C.

Regarding the other compound classes (e.g., peptides and carbohydrates), each of these classes is composed of known chemical structures with similar backbone configurations. Therefore, we adopted the single ratio provided by Anderson (1995) and further explained in Fox et al. (2018).

Supplementary Figures:

Figure S1: Comparison of ^{1}H-NMR spectra of total seawater and SPE (PPL) DOM. Sample spectra from the Canadian Arctic Archipelago are shown for (A) station C013 40 m, and (B) station 518 0 m. For both plots, orange represents total seawater spectra obtained using the watergate method, blue and grey represent DOM isolated by PPL extraction using poly(divinylbenzene-co-N-vinylpyrrolidone) resin measured by watergate and traditional D_2O ^{1}H-NMR, respectively.

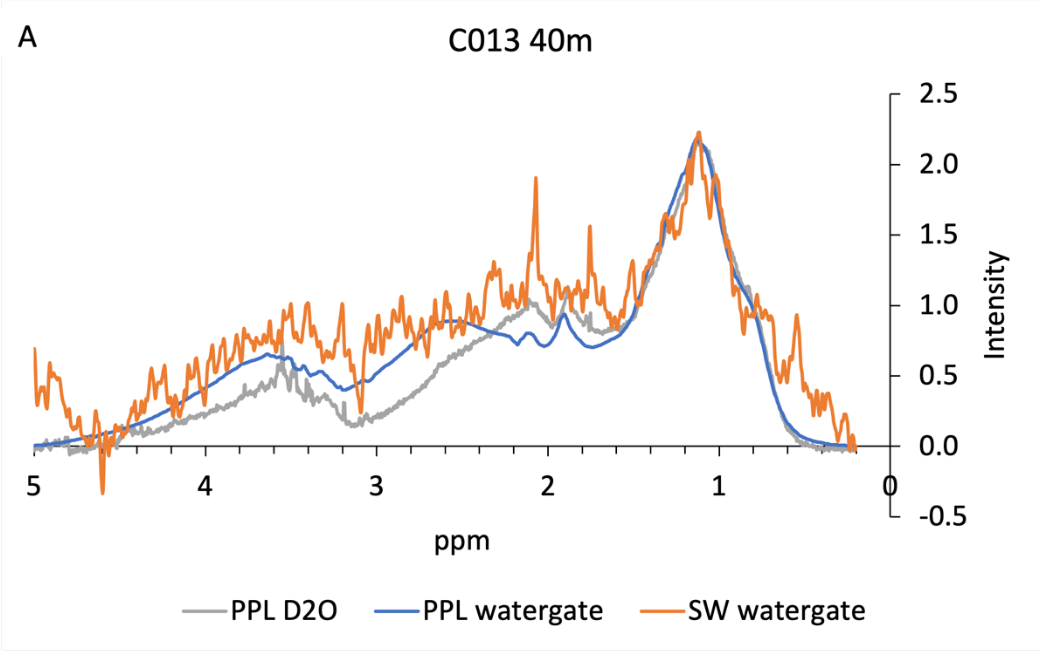

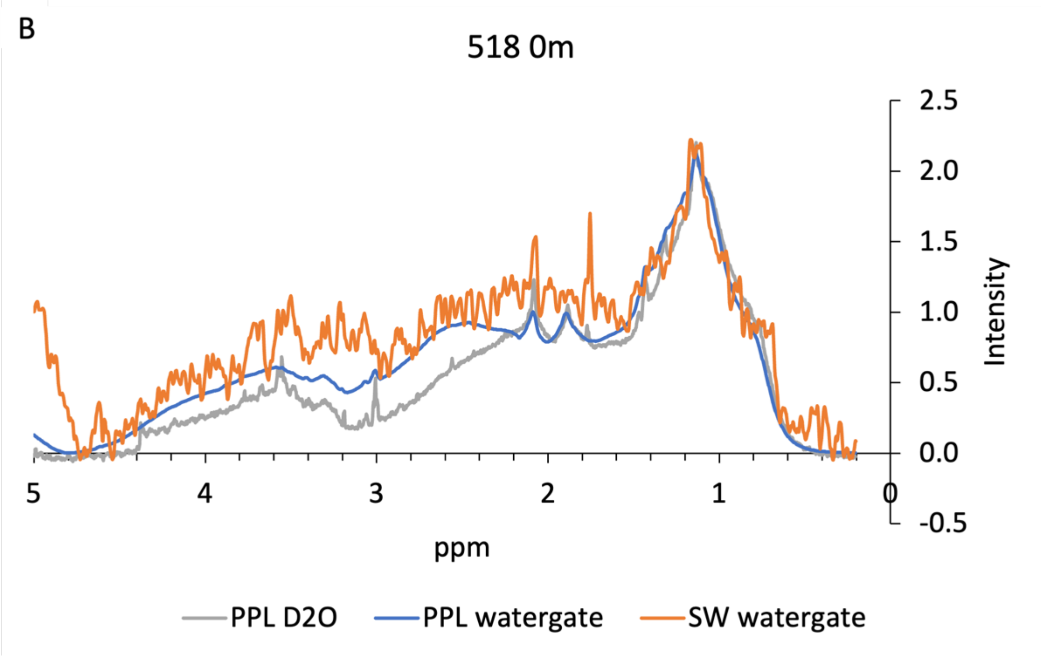

Figure S2: Section plots following the two major current systems in Baffin Bay; West Greenland Current (WGC: A, B) and Baffin Island Current (BIC: C, D). The colour map indicates the percent abundance of each of the compound classes; CRAM (A, C), or carbohydrates (B, D). The black circles show the location where each sample was taken. Black regions represent the bathymetry of the seafloor.

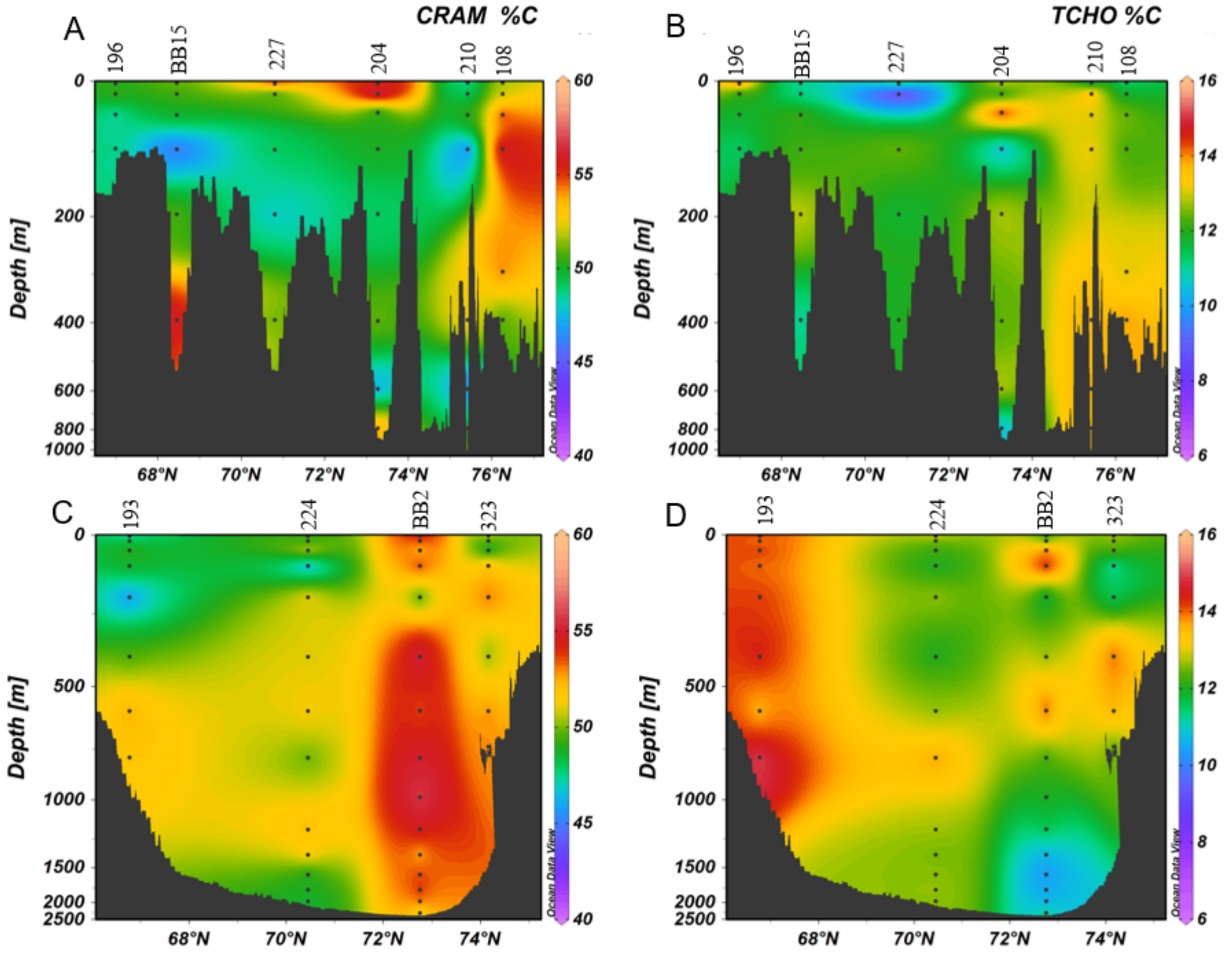

Figure S3: Depth profiles from all stations. (A, B) Percent relative abundance of CRAM and TCHO respectively. (C, D, E) Concentration of CRAM, TCHO and DOC, respectively. Station specific changes in %TCHO are observed within a few water masses. High concentrations of BIC [DOC] (Stations 193, 224; 0-250 m. Figure 2F and S2C) co-occur with high %TCHO (Figure S2D), perhaps indicating export of DOM with unique chemical composition, largely modified by Pacific and Canadian Arctic Archipelago source contributions, to the Labrador Sea. High %TCHO within the nepheloid layer of Station BB2 (13.4%) relative to overlying deep water (average = 10% TCHO from 1400-2000 m) is a strong indication of new labile DOM being added to Baffin Bay Deep Water (BBDW >2000 m).

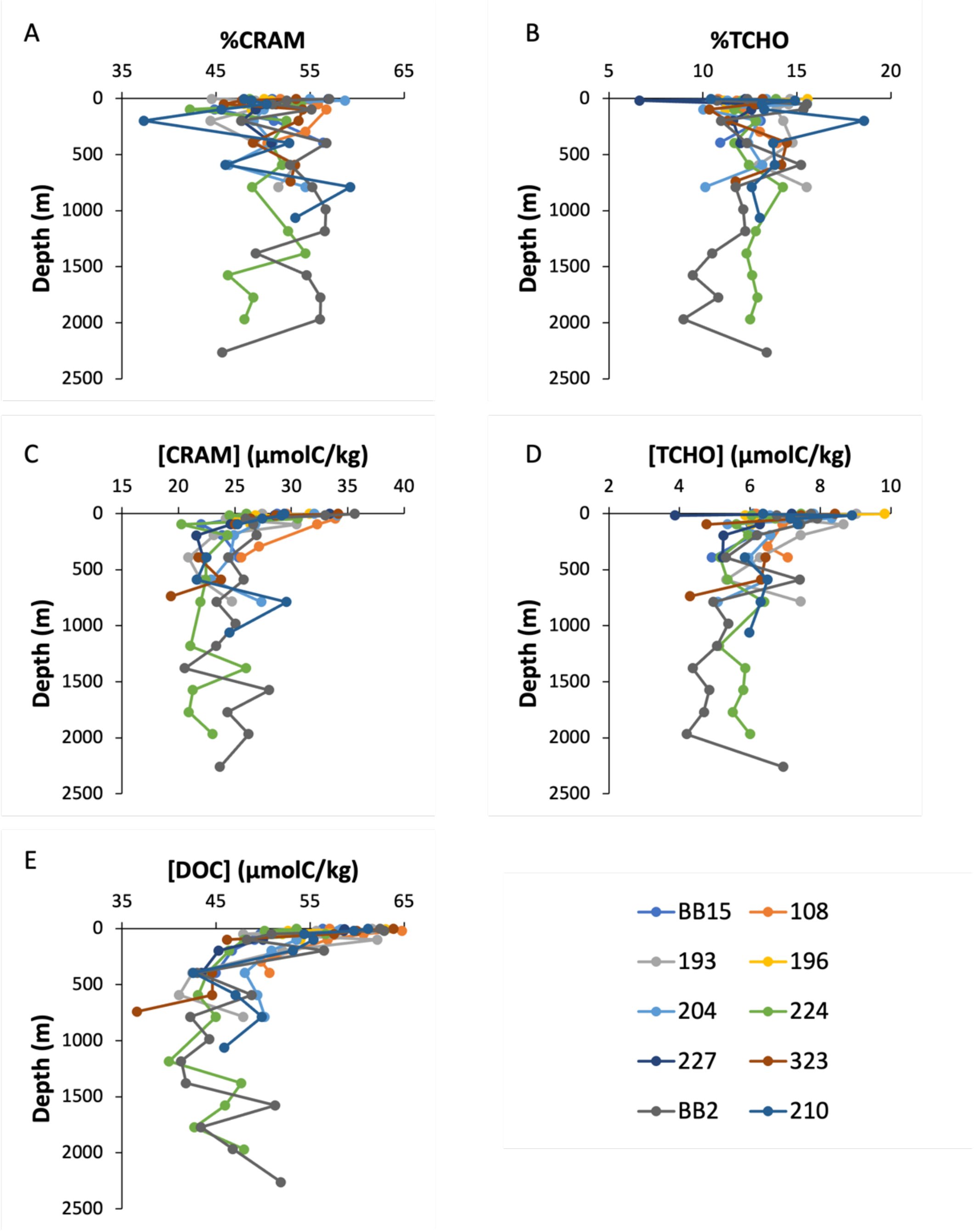

Figure S4. Sections of chromophoric dissolved organic matter (CDOM) in Baffin Bay. CDOM data shown was recorded using a Seabird Wetlabs ECO Fluorometer (FLCDRTD-2344) with DOM fluorescence measured at Ex/Em of 370/460 nm. Sections were generated in Ocean Data View for Lancaster Sound (323), Smith Sound (108; 115), Central Baffin Bay (224; 227) and Davis Strait (193; 196). A decrease in the CDOM maximum (~100 m depth) is observed along the nearshore Baffin Island Current extension (<100 km from shore) presumably due to mixing/dilution.

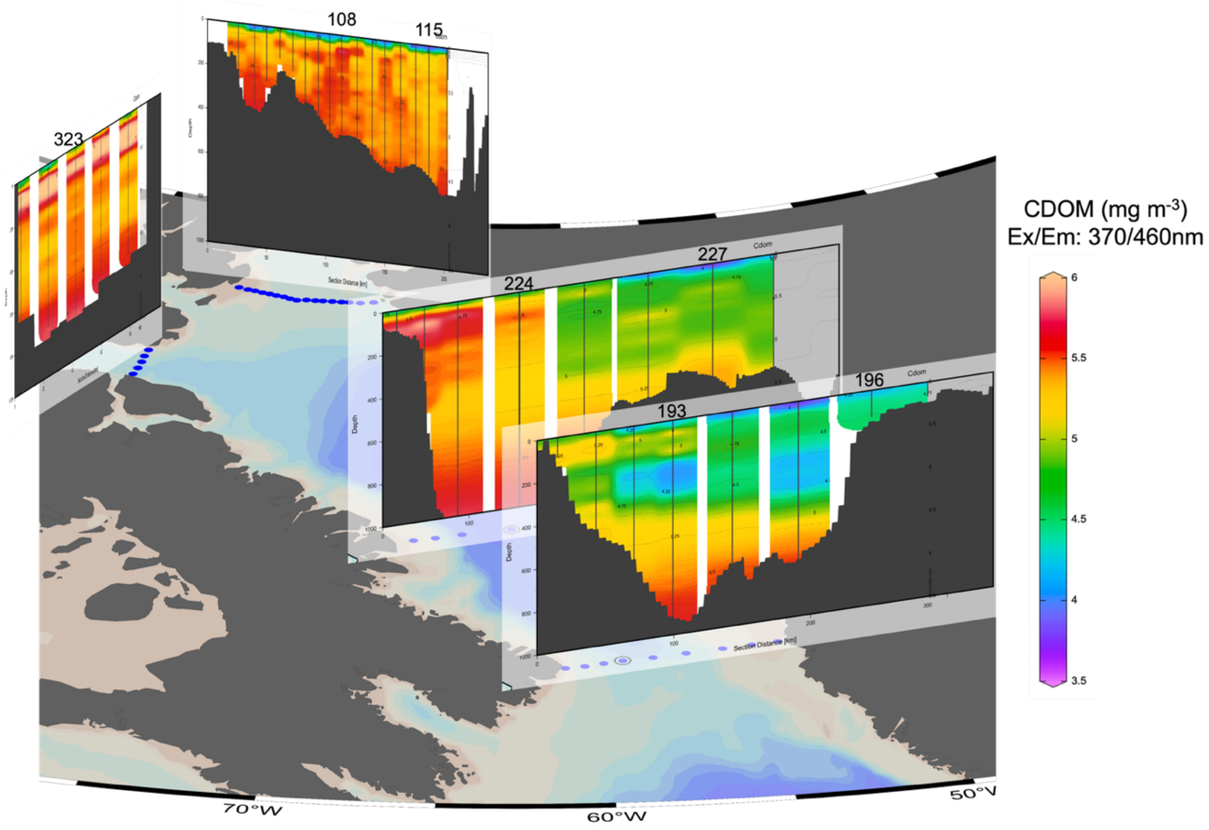

Figure S5: Example spectrum with shaded regions showing the integration areas of major compound classes. Major compound classes were identified via principal component analysis of all 91 samples collected following Fox and co-workers (2018)[20]. The green areas include CHO, CHO-deoxysugar, CHO-carbohydrates, N-acetyl amino sugar, and CH_3-deoxysugar, were summed to compute the integrated area of TCHO. The area used to calculate CRAM is shaded in red. The compound classes that were not discussed in this study include molecularly uncharacterized aliphatics (MUCA) shaded in orange, as well as methanethiol, proteins & peptides, and alkyls shaded in blue.

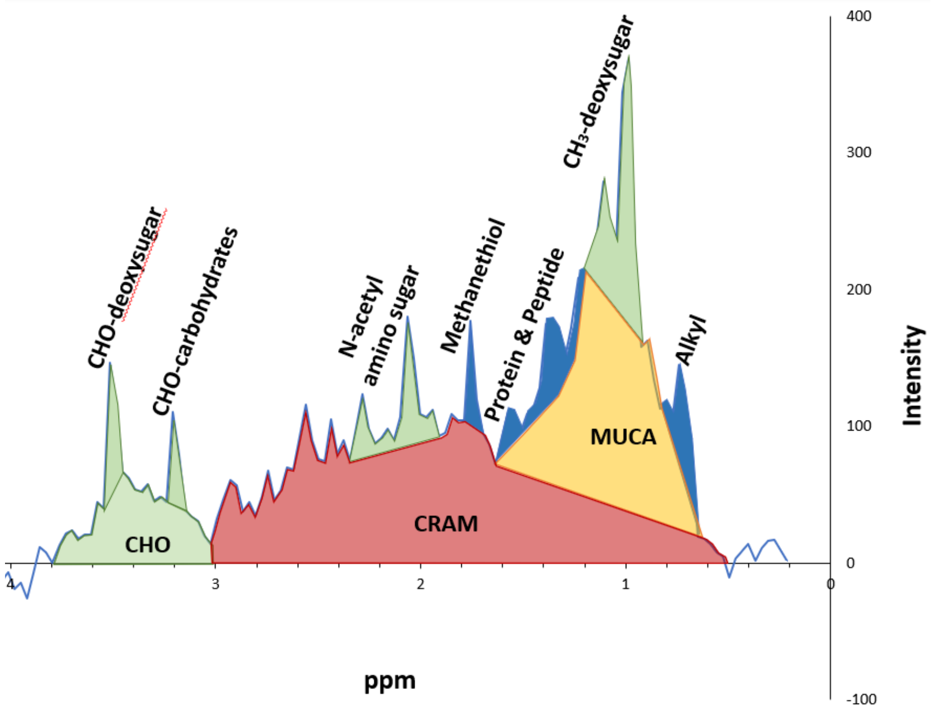

Supplementary Tables:

Table S1: Summary of Results – Comparison of ¹H-NMR spectra of total seawater and SPE (PPL) DOM %CRAM, %TCHO and [CRAM], [TCHO] composition. Total seawater and SPE DOM in water were analyzed using the "watergate" water suppression NMR technique. SPE DOM in D_2O was analyzed as traditional D_2O ¹H-NMR experiments.

Sample ID	Yield (%DOC)	CRAM (%C)	TCHO (%C)	[DOC] (µmol C kg^{-1})	[CRAM] (µmol C kg^{-1})	Difference vs SPE watergate [CRAM]	[TCHO] (µmol C kg^{-1})	Difference vs SPE watergate [TCHO]	[CRAM]: [TCHO] Ratio	Difference vs SPE watergate [CRAM]: [TCHO] Ratio
**Total Seawater:**										
210 800 m	100%	59.3	12.6	49.9	29.6	52%	6.3	52%	4.72	1%
518 0 m	100%	56.5	14.6	84.9	47.9	58%	12.4	62%	3.87	-10%
C010 37.5 m	100%	59.0	13.7	102.2	60.3	65%	14.0	70%	4.32	-16%
C013 40 m	100%	61.3	14.9	76.9	47.1	60%	11.4	59%	4.12	1%
**SPE DOM in H2O:**										
SPE 210 800 m	55%	51.8	11.1	27.4	14.2	-	3.0	-	4.68	-
SPE 518 0 m	45%	52.8	12.4	38.2	20.2	-	4.7	-	4.26	-
SPE C010 37.5 m	39%	53.6	10.7	39.9	21.4	-	4.3	-	5.02	-
SPE C013 40 m	49%	50.2	12.4	37.7	18.9	-	4.7	-	4.06	-
SPE MeOH blank	-	28.2	19.6	0.0	0.0	-	0.0	-	-	-
**SPE DOM in D2O:**										
SPE 518 0 m	45%	52.2	10.1	38.2	20.0	-1%	3.9	-22%	5.15	17%
SPE C013 40 m	49%	52.8	8.8	37.7	19.9	5%	3.3	-40%	5.98	32%
SPE MeOH blank	-	24.7	14.6	0.0	0.0	-	0.0	-	-	-

Table S2: List of major ^{1}H NMR compound classes and corresponding chemical shift (d_H) ppm values from sediment pore water (adapted from Fox et al., 2018)[20]. The ppm range corresponds to the chemical shift of the measured proton. The assignment indicates the position of the measured proton within the compounds functional group. The compound class is a broad classification that encompasses many types of similar molecules.

d_H (ppm) [c]	H:C	**H**-Assignment	Compound Class [c]
0.68-0.84	3.00	$C\mathbf{H}_3X$	Alkyl (Methyl)
0.84-1.24	1.67[a]	$C\mathbf{H}_3$ in 6-deoxysugars[d]	Carbohydrates
1.24-1.65	1.58 [a]	$C\mathbf{H}_2$-C-CO(NHR) [c]	Protein and peptides
1.70-1.84	2.00 [a]	CH_3-C-S**H** [c]	Methanethiol
1.95-2.22	1.67 [a]	CH_3-C=O-N**H**[e]	N-acetyl amino sugars; acetate derivatives
2.72-2.80	1.67 [a]	CH_3-CH_2-C=O-N**H** [c]	Amino sugars
2.94-3.15	1.58 [a]	CH_2-N$\mathbf{H}_2$ [c]	Protein and peptides
1.51-1.64	1.00[b]	**H**C(C)-COX [b]	Subsection of CRAM
2.22-2.72	1.00 [b]	**H**C(C)-COX [b]	Subsection of CRAM
0.91-3.15	1.00 [b]	**H**C(C)-COX [b]	CRAM
3.15-4.14	1.67 [a]	**H**C-O [c]	Carbohydrates

[a]H:C ratios of major biochemical classes of Anderson (1995)[21] were assigned
[b]H:C ratio and H-assignment data adapted from Hertkorn (2013)[1]
[c]Fox (2018)[20]
[d]Quan and Repeta (2007)[22]
[e]Aluwihare et al. (2005)[23]; Hertkorn et al. (2013)[1]

Table S3: Abundance (%) and Concentration (µmol C kg^{-1}) of major compound classes as well as standard deviation (SD) in duplicate (DUP) samples. Duplicates represent samples prepared for NMR analysis from separate ampules collected from the same Niskin bottle at sea. Dates of NMR preparation and analysis are listed below each duplicate number as yyyy/mm/dd.

			Alkyl (methyl) (R-CH_3)	CH_3-deoxysugar	CH_3-deoxysugar	Protein & peptide	Amine / peptide	Protein & peptide	Methanethiol	MUCA	CHO-deoxysugar (O-methylrhamnose / O-methylfucose)	CRAM	CHO-carbohydrates	TCHO
		ppm	0.68-0.84	0.84-1.05	1.05-1.24	1.24-1.31	1.31-1.45	1.45-1.65	1.70-1.84	0.68-1.65	3.44-3.56	0.91-3.15	3.15-4.14	0.93-1.24, 3.15-4.14
**Station 193 100 m**	Abundance (%)	DUP1 2020/06/09	0.35	1.21	1.25	0.58	0.69	0.47	0.39	26.37	1.46	49.26	8.67	13.20
		DUP2 2020/10/14	0.36	0.60	1.18	0.22	0.66	0.27	0.37	23.47	1.36	48.98	10.12	13.90
		**SD**	**0.01**	**0.43**	**0.05**	**0.26**	**0.02**	**0.14**	**0.01**	**2.05**	**0.07**	**0.20**	**1.02**	**0.49**
	Concentration (µmol kg^{-1})	DUP1 2020/06/09	0.22	0.75	0.78	0.36	0.43	0.29	0.24	16.41	0.91	30.65	5.40	8.22
		DUP2 2020/10/14	0.23	0.37	0.74	0.13	0.41	0.17	0.23	14.60	0.84	30.48	6.30	8.65
		**SD**	**0.01**	**0.27**	**0.03**	**0.16**	**0.01**	**0.09**	**0.01**	**1.28**	**0.20**	**0.12**	**0.09**	**0.30**
**Station BB2 0 m**	Abundance (%)	DUP1 2020/07/28	0.15	1.17	0.90	0.29	3.15	0.38	0.22	15.22	1.20	57.04	8.88	12.36
		DUP2 2020/03/13	0.25	0.44	1.21	0.27	0.57	0.37	0.30	25.31	0.77	52.20	10.90	13.42
		DUP3 2021/02/06	0.12	0.59	1.13	0.12	0.79	0.15	0.47	19.59	1.23	52.89	9.01	12.30
		**SD**	**0.07**	**0.39**	**0.16**	**0.09**	**1.43**	**0.13**	**0.13**	**5.06**	**0.26**	**2.62**	**1.13**	**0.63**
	Concentration (µmol kg^{-1})	DUP1 2020/07/28	0.09	0.73	0.56	0.18	1.97	0.23	0.14	9.51	0.75	35.66	5.55	7.73
		DUP2 2020/03/13	0.15	0.28	0.76	0.17	0.36	0.23	0.19	15.82	0.48	32.63	6.81	8.39
		DUP3 2021/02/06	0.08	0.37	0.71	0.08	0.50	0.09	0.30	12.25	0.77	33.06	5.63	7.69
		**SD**	**0.04**	**0.24**	**0.10**	**0.06**	**0.89**	**0.08**	**0.08**	**3.16**	**0.16**	**1.64**	**0.71**	**0.39**

Supplementary References:

1. Hertkorn, N., Harir, M., Koch, B. P., Michalke, B. & Schmitt-Kopplin, P. High-field NMR spectroscopy and FTICR mass spectrometry: powerful discovery tools for the molecular level characterization of marine dissolved organic matter. *Biogeosciences* **10**, 1583–1624 (2013).

2. Broek, T. A. B., Walker, B. D., Guilderson, T. P. & McCarthy, M. D. Coupled ultrafiltration and solid phase extraction approach for the targeted study of semi-labile high molecular weight and refractory low molecular weight dissolved organic matter. *Mar Chem* **194**, 146–157 (2017).

3. Broek, T. A. B. *et al.* Low Molecular Weight Dissolved Organic Carbon: Aging, Compositional Changes, and Selective Utilization During Global Ocean Circulation. *Global Biogeochem Cy* **34**, (2020).

4. Zeidan, S. *et al.* Using Radiocarbon Measurements of Dissolved Inorganic Carbon to Determine a Revised Residence Time for Deep Baffin Bay. *Frontiers Mar Sci* **9**, 845536 (2022).

5. Fox, A. & Walker, B. D. Sources and Cycling of Particulate Organic Matter in Baffin Bay: A Multi-Isotope δ^{13}C, δ^{15}N, and Δ^{14}C Approach. *Front. Mar. Sci.* **9**, 846025 (2022).

6. Münchow, A., Falkner, K. K. & Melling, H. Baffin Island and West Greenland Current Systems in northern Baffin Bay. *Prog Oceanogr* **132**, 305–317 (2015).

7. Curry, B., Lee, C. M. & Petrie, B. Volume, Freshwater, and Heat Fluxes through Davis Strait, 2004–05*. *J Phys Oceanogr* **41**, 429–436 (2011).

8. Hamilton, J. & Yongsheng, W. *Synopsis and Trends in the Physical Environment of Baffin Bay and Davis Strait*. (Department of Fisheries and Oceans. Maritimes Region.; Bedford Institute of Oceanography, 2013).

9. Münchow, A., Falkner, K. K. & Melling, H. Baffin Island and West Greenland Current Systems in northern Baffin Bay. *Prog. Oceanogr.* **132**, 305–317 (2015).

10. Melling, H., Gratton, Y. & Ingram, G. Ocean circulation within the North Water polynya of Baffin Bay. *Atmos.-Ocean* **39**, 301–325 (2001).

11. Tang, C. C. L. *et al.* The circulation, water masses and sea-ice of Baffin Bay. *Prog. Oceanogr.* **63**, 183–228 (2004).

12. Shiklomanov, A. *et al.* Arctic Hydrology, Permafrost and Ecosystems. 703–738 (2020) doi:10.1007/978-3-030-50930-9_24.

13. Azetsu-Scott, K., Petrie, B., Yeats, P. & Lee, C. Composition and fluxes of freshwater through Davis Strait using multiple chemical tracers. *J. Geophys. Res.: Oceans* **117**, (2012).

14. Mungall, E. L. *et al.* Microlayer source of oxygenated volatile organic compounds in the summertime marine Arctic boundary layer. *Proc. Natl. Acad. Sci.* **114**, 6203–6208 (2017).

15. Burgers, T. M. *et al.* Surface Water pCO_2 Variations and Sea-Air CO2 Fluxes During Summer in the Eastern Canadian Arctic. *J. Geophys. Res.: Oceans* **122**, 9663–9678 (2017).

16. Lalande, C., Forest, A., Barber, D. G., Gratton, Y. & Fortier, L. Variability in the annual cycle of vertical particulate organic carbon export on Arctic shelves: Contrasting the Laptev Sea, Northern Baffin Bay and the Beaufort Sea. *Cont. Shelf Res.* **29**, 2157–2165 (2009).

17. Lehmann, N. *et al.* Remote Western Arctic Nutrients Fuel Remineralization in Deep Baffin Bay. *Global Biogeochem Cy* **33**, 649–667 (2019).

18. Shen, Y., Benner, R., Kaiser, K., Fichot, C. G. & Whitledge, T. E. Pan-Arctic Distribution of Bioavailable Dissolved Organic Matter and Linkages With Productivity in Ocean Margins. *Geophys Res Lett* **45**, 1490–1498 (2018).

19. Guéguen, C., Cuss, C. W., Cassels, C. J. & Carmack, E. C. Absorption and fluorescence of dissolved organic matter in the waters of the Canadian Arctic Archipelago, Baffin Bay, and the Labrador Sea. *J. Geophys. Res.: Oceans* **119**, 2034–2047 (2014).

20. Fox, C. A., Abdulla, H. A., Burdige, D. J., Lewicki, J. P. & Komada, T. Composition of Dissolved Organic Matter in Pore Waters of Anoxic Marine Sediments Analyzed by 1H Nuclear Magnetic Resonance Spectroscopy. *Frontiers Mar Sci* **5**, 172 (2018).

21. Anderson, L. A. On the hydrogen and oxygen content of marine phytoplankton. *Deep Sea Res Part Oceanogr Res Pap* **42**, 1675–1680 (1995).

22. Quan, T. M. & Repeta, D. J. Periodate oxidation of marine high molecular weight dissolved organic matter: Evidence for a major contribution from 6-deoxy- and methyl sugars. *Mar Chem* **105**, 183–193 (2007).

23. Aluwihare, Repeta, D., Pantoja, S. & Johnson, C. Two Chemically Distinct Pools of Organic Nitrogen Accumulate in the Ocean. *Science* **308**, 1007–1010 (2005).
