# Supplementary material for: Cycling of labile and recalcitrant carboxyl-rich alicyclic molecules and carbohydrates in Baffin Bay
Source: Nat Commun. 2024 Oct 9;15:8735. doi: 10.1038/s41467-024-53132-5 (PMC11464691; doi:10.1038/s41467-024-53132-5)
Supplement: Supplementary file 2 — Description of Additional Supplementary Files [file 41467_2024_53132_MOESM2_ESM.pdf]

Description of Additional Supplementary Files

File Name: Supplementary Data 1
Description: Summary of hydrographic, DOC and compound-class information for all stations.
