# Supplementary material for: Cycling of labile and recalcitrant carboxyl-rich alicyclic molecules and carbohydrates in Baffin Bay
Source: Nat Commun. 2024 Oct 9;15:8735. doi: 10.1038/s41467-024-53132-5 (PMC11464691; doi:10.1038/s41467-024-53132-5)
Supplement: Supplementary file 4 — Source Data [file 41467_2024_53132_MOESM4_ESM.pdf]

nature portfolio

Peer Review File

Cycling of labile and recalcitrant carboxyl-rich alicyclic molecules and carbohydrates in Baffin Bay

Corresponding Author: Professor Brett Walker

This file contains all editorial decision letters in order by version, followed by all author rebuttals in order by version.

Version 0:

Reviewer comments:

Reviewer #1

(Remarks to the Author)
McKee et al.
Cycling of bioavailable carboxyl-rich alicyclic molecules and carbohydrates in Baffin Bay

The complexity of chemical structures of dissolved organic matter (DOM) has limited our understanding of the exact pathways of carbon cycling in the ocean. McKee et al. used a new method to quantify the concentrations of total carbohydrates (considered to be labile) and CRAM (considered to be recalcitrant) using samples collected from the Baffin Bay in the Canadian Arctic. The authors observed degradation of the labile as well as recalcitrate components with depth in the Baffin Bay, challenging the traditional view that CRAM should be more persistently present in deeper water.

The value of this manuscript is the development of a novel method using total seawater DOM proton (1H) nuclear magnetic resonance (NMR) spectroscopy, which significantly enhanced the recovery of DOM, leading to the quantification of CRAM degradation in deeper water of up to 34% of its production in the surface ocean. This finding is profound and may change our view of CRAM as a refractory marker for DOM cycling in the ocean. However, I found the authors to sell themselves short in their claims, which raise questions that must be answered before people can adapt this method for improving our understanding of the mechanisms of DOM degradation in the ocean.

My first question is the comparison of the methods. The authors claimed that the traditional salt-free DOM extraction methods (ultrafiltration and/or solid-phase extraction) can only recover less than 20-40% of total DOM, which also induce chemical and isotopic biases that impede the characterization of DOM compositions. It is desirable that the authors perform these traditional analyses for comparison with the total seawater DOM proton (1H) NMR method, rather than relying on literature reports.

That leads to my second question: How would the DOM chemical structures differ between NMR, ultrafiltration and solid-phase extraction methods? This question should be answered by performing all three types of analyses using the same sets of samples. I did not find these analyses in the method section.

My next question would be the actual mechanism for the degradation of CRAM in deeper water of the Baffin Bay. Although the authors use the word “bioavailable” in the title, implying microbial degradation of CRAM, no experimental data were provided. This question should be answered by performing cultivation experiments as in Zheng et al. (2022) that was cited by this manuscript.

The authors may have a wonderful opportunity to test a hypothesis about CRAM degradation enhanced by the priming effect of more labile DOM (carbohydrates or proteins) accompanying CRAM going down the water column in the Baffin Bay. This would make the paper much stronger by telling a compelling story of the mechanisms of DOM degradation rather than a simple report of what happened (the decrease in CRAM abundance with depth).

The authors should also provide more thorough information of the unique environment of the Baffin Bay: the terrestrial input of nutrients to the seawater in Baffin May, the impact of land-derived organic matter on the complexity of DOM in Baffin Bay, and the production of phytoplankton contributing to the fresh labile DOM in your samples.

Other comments are line-by-line:

Line 42: “The Arctic Ocean..” should be a new paragraph.
Line 55: “It was inferred..” should be a new paragraph.
Line 62: In “UDOM”, what is U representing?
Line 106: This paragraph should move up above line 100.
Line 111: 100m, 200m: Should be “100 m, 200 m.” The same correction is needed in many other places.
Line 115: Spell out “MBTH”.
Lines 118-119: The sentence “This work is beyond..” is distractive. Suggest to delete.
Line 124: How does this “missing” CRAM differ in chemical structure from UF- or SPE-derived CRAM ? A PCA analysis should be good.
Line 143 and many other places: A space is needed before and after the “=” sign.
Lines 161-162: Is it meaningful to report the p value as 0.0000028 or 0.000000041. Should the p value < than 0.001 be enough?
Lines 178-179: “This CRAM is biologically available and rapidly removed in the surface ocean”. It will be good to support this claim with experimental data.
Lines 213-215: The statement here is too speculative.
Lines 220-222: The statement of 14C age is too much speculation.

Figures 1B and 1C: The temperature and salinity impact on DOM composition between stations on ether side of the Baffin Bay should be discussed.
Line 251 and other places: 50um should have a space in between.
Line 452 and other places: 2mL should have a space in between.
Line 447 and other places: 4.8ppm should have a space in between.

In summary, I like the way the paper is trying to accomplish, but the story needs to be beefed up tremendously. I would very much like the story to be fully developed taking consideration of my comments and suggestions. At this stage, it does not meet the requirement for publication in NC.

Reviewer #2

(Remarks to the Author)
Dear Editor,

Sub: Review of a manuscript (NCOMMS-23-27002-T) entitled “Cycling of bioavailable carboxyl-rich alicyclic molecules and carbohydrates in Baffin Bay” by McKee et al.

While the study presents interesting findings related to the cycling and estimation of the quantities of two major structural groups of dissolved organic matter (DOM) - total carbohydrates and carboxyl-rich alicyclic molecules (CRAM) in Baffin Bay - and proposes a two-pool CRAM model, I regret to inform you that the manuscript, in its current form, falls short of the standards expected for publication in Nature Communications. The following concerns underlie my recommendation:
The authors have employed a previously published total seawater 1D 1H NMR technique to study the cycling of DOM, which is advantageous for its comprehensive analysis without the need for a preconcentration step and avoidance of chemical or size-based extraction biases. However, the well-known limitations of poor resolution and peak attenuation around the water signal raise questions about the accuracy of the quantitative analysis of DOM structural groups, such as total carbohydrates and CRAM. Consequently, I recommend that the authors validate their findings using complementary analytical approaches, such as high-resolution mass spectrometry.
While this study demonstrates that not all CRAM is recalcitrant, but a fraction of the CRAM pool of DOM is biologically labile, it is essential to reinforce these conclusions through robust evidence. I urge the authors to undertake additional experiments or analyses to confirm and strengthen the proposed two-pool CRAM model, ensuring the reliability of their results.

Specific comments and questions:
1. 1D 1H NMR suffers from poor spectral resolution due to the spread of H-H J-coupling multiplets over a limited chemical shift range of ~10 ppm, particularly for highly intricate molecular mixtures such as DOM. Additionally, the 600 MHz NMR employed in this study is not an optimal instrument for resolving the signals from millions of chemically distinct compounds present in DOM and improving the detection of signals from very dilute compounds. The 1H NMR integration region of CRAM (3-0.5 ppm) chosen in this study may have a significant contribution from the protons of other structural groups of DOM due to overlapping. How did the authors account for this bottleneck? How confident are the authors about quantifying exclusively the CRAM fraction of DOM in the highly crowded 1H NMR spectra?
2. To confirm the 1D 1H NMR-based findings of this manuscript, it would be beneficial to have complementary evidence, such as ultrahigh-resolution FT-ICR mass spectrometry analysis. Unfortunately, 2D NMR analysis is not feasible due to the extremely low concentration of the samples.
3. How distinct are the two CRAM fractions (recalcitrant vs. labile) of DOM structurally? What factors make a specific fraction of CRAM biodegradable? Is the labile CRAM fraction only biodegradable, or is it also photodegradable?
4. When using the 1H NMR pulse sequence reported by Lam et al. (Analyst, 2008, 133, 263–269), signals up to 1.1 ppm on each side of the water signal (in the range approximately 5.9-3.7 ppm, considering the water signal appeared at 4.8 ppm) will be attenuated, which drastically affects the quantification of carbohydrates. The authors should clarify how accurate their quantification of total carbohydrates is under these conditions.
5. Figures: There are only two figures in the main text, one of which (Figure 1) is adapted from an already published paper.

Since this is an original research article and not a review, the adapted figure does not add any new information to strengthen the manuscript. I understand that it is important to show the location of the sampling sites, so the authors should consider providing this figure in the Supplementary Information and using the space in the main text more effectively. Additionally, it is challenging to read the scale in Figure 2; therefore, the resolution and font size should be increased for better visibility.
6. The findings of this manuscript are mainly based on the 1D 1H NMR of total seawater; however, the 1H NMR spectra are not shown in the manuscript, not even in the Supplementary Information. To evaluate the authors conclusions, a comparison of 1H NMR spectra should be provided as a figure, at least in the Supplementary Information.
7. Lengthy abstract: The abstract contains approximately 273 words, which is much longer than the 150-word limit recommended by the journal.
8. Lines 15-26: Literature should not be cited in the abstract.
9. Lines 22-24: The statement regarding the solid-phase extraction efficiency (<20-40%) is not correct. The solid-phase (PPL) extraction efficiency for DOM has been greatly improved over the past decade, and the recovery rate for both deep and surface ocean bulk DOM was 61%. (Reference: Green et al., Marine Chemistry, 2014, 161, 14-19; An intercomparison of three methods for the large-scale isolation of oceanic dissolved organic matter).
10. Line 75: It is “mass spectrometry” but not “mass spectroscopy”.
11. Lines 75-76: The abbreviation of CRAM is "carboxyl-rich alicyclic molecules," not "carboxyl-rich alicyclic material."
12. Line 116: Please replace "From our integrations" with “Based on 1H NMR integrations”.
13. Line 126: The statement regarding the SPE-DOC recovery (~40%) is not correct. Please refer to comment 9.
14. Line 212: SPE-DOM is not a technique, but it is the fraction of DOM extracted by the SPE technique.
15. Line 257: Please replace “was” with “were”
16. Lines 262 and 500: “dH” denotes the enthalpy change in thermodynamics. The chemical shift is denoted by the symbol "δ" but not "d". Please replace "dH" with "δH" to denote proton chemical shift and ensure that it is corrected throughout the main text and supplementary information, wherever applicable.
17. Line 474: Please replace “was” with “were”
18. Line 475: TMS (tetramethylsilane) is a good NMR internal standard for most organic solvents, but it has very low solubility in water and is not generally used as an internal reference in aqueous solutions. Since all the spectra were recorded in water in this study, the authors should clarify the use of TMS as an NMR internal standard instead of DSS (sodium 4,4-dimethyl-4-silapentane-1-sulfonate) or DSA (4,4-dimethyl-4-silapentane-1-ammonium trifluoroacetate) which are preferred NMR standards for water samples. Did the authors intentionally add TMS to the NMR sample, or did they mistakenly assume naturally occurring silicate species as TMS?
19. “Results and Discussion” section heading is missing in the main text.
20. The authors should provide a complete description of 1H NMR experimental and processing parameters at least in the Supplementary Information to ensure reproducibility.
21. Data availability statement: The data availability was not indicated. It is necessary to deposit time-domain and processed NMR data that support the findings of this manuscript to repositories to ensure reproducibility.

Reviewer #3

(Remarks to the Author)
McKee et al. present an interesting study that suggests a “labile” CRAM pool in the surface ocean, contrary to its typical classification as a major component of the “refractory” DOM pool. The concept of 1H NMR analysis on whole water samples is also highly interesting, and results of this study highlight its potential to re-evaluate our understanding of marine DOM composition beyond what we have learned from analyses of SPE-DOM. That said, I would have appreciated more details on the methodology, particularly in the calculation of carbon-based concentrations from 1H NMR data. Perhaps this could be supplied in the SI, with PCA analyses. I also wonder how these results compare to “% CRAM” results determined from 13C NMR (or is it 2D NMR?). e.g., Lines 52-55 state, "Through 13 C-NMR measurements it was estimated that ~50% of surface ultrafiltered high molecular weight DOM (HMW DOM; >1000 Da) was carbohydrates whereas both surface and deep HMW DOM were composed of primarily aliphatic molecules and carboxylic acids14,15. “ Moreover, lines 72-72 reiterate “Based off 13 C-NMR, this material is found throughout the water column, but in higher relative total abundance in deep UDOM, suggesting a refractory “humic” background DOM component that lasts several oceanic mixing cycles15,21.” I imagine there is a carbon limitation here, so only 1H NMR is possible without preconcentrating DOM, but I wonder if there are large differences between calculated %CRAM using this approach and that from Hertkorn et al. 2013. I also wonder if Hertkorn et al. (2013) suggest 51% (5 m) - 56% (5446 m) of SPE-DOM as CRAM, wouldn’t the same trend be observed as this study, if DOC extraction efficiencies do not change with depth (as suggested by Ksionzet et al. 2016, Science(354), doi: 10.1126/science.aaf7796)? There will be more SPE-DOC in the surface than at depth, and 51% vs. 56% CRAM would mean higher CRAM concentrations in the surface? All this is to say that these additional details/answers to these questions may help explain why the results obtained here are unique and why they suggest that CRAM in the surface exists in at least 2 pools (labile and refractory). Additional details should also help explain why the %TCHO and %CRAM calculations are so noisy.

Another thought: if this work suggests that CRAM is not as refractory as previously suggested, couldn’t this work also imply that TCHO is not as labile as previously thought?

Minor comments listed below:
Line 115: what is MBTH?
Lines 157-159: Are these average values that are reported, like those reported for %TCHO and %CRAM? What is the standard deviation? It does seem like there is a lot of variability (Figure S2), so perhaps could help in understand how these values are significantly different (apart from the ANOVA that is discussed next).
Lines 161-163: I would argue that a decrease in 1 umol/kg by NMR vs 3 umol/kg by MBTH is not consistent, but rather a

large difference, especially if the decrease is from 7 to 6 umol/kg (NMR) vs 7 to 4 umol/kg (presumably by MBTH).
Figure 2: the scales on the colour bars could be larger, it is hard to tell if West Greenland Current data and Baffin Island Current data are plotted on the same scale.
Figure S2: the labels are quite small and difficult to read. Any error associated with these points? I would also appreciate seeing the DOC data plotted in this manner (not just ODV plots).
Table S2 could also benefit from larger font.
Line 250: Is centrifugation necessary with filtered samples?
Line 432: define KHP.

Version 1:

Reviewer comments:

Reviewer #1

(Remarks to the Author)
It is obvious that the authors tried hard to address my previous comments, with some answers satisfying. But my key concern or request for ground truth was not satisfied. Particularly these two questions:

1. The underlying mechanism allowing the total DOM proton (1 H) NMR technical to be superior to ultrafiltration or SPE, which were claimed to induce chemical-, size- and isotopic-bias. Will the novel method remove these biases?

2. It is thus contradicting to state that "it is plausible SPE isolates a similar CRAM population (and concentrations)" while the new method covers greater abundance of CRAM with the same chemical structures.

Based on Figure S5 and Table S2, would it be possible to do a linear discriminant analysis (LDA) or similar comparison analysis to show what chemical compounds are distinctive or as the authors claimed the novel method recovers CRAM populations having the same composition as the SPE.

Overall, there are more questions arising than what the revised paper can answer.

There are many typos in the main text and supplemental information the authors need to correct, which are highlighted in their PDF documents I submitted.

Reviewer #2

(Remarks to the Author)

Reviewer #3

(Remarks to the Author)
I still believe that the manuscript is novel and appropriate for publication in Nature Communications. I also appreciate the new information using SPE-DOM. However, I am still unsure as to the validity and error associated with the conversion to carbon content/concentrations given in the section on spectral integration of compound classes. The authors do state in their response that additional details are given, but without line numbers and track changes they are hard to follow - inspecting both the revised and the original manuscript I cannot find these details (now lines 402-424). This conversion appears to come from a paper on H and O content in phytoplankton (ref 43) using H/C ratios table S3. Are there not larger ranges in the H/C ratios for the compound classes reported elsewhere (or as FT ICR MS data may suggest)? Is H/C = 1 used for CRAM (Table S3)? I would a appreciate a bit more discussion on this point so we can know if the significant decreases in CRAM (3 umol C/kg) with depth (line 194) are truly significant, or just in the noise. Otherwise, I am satisfied with the review response and revisions.

Version 2:

Reviewer comments:

Reviewer #3

(Remarks to the Author)
I am satisfied with author comments and recommend this article for publication. I only have one last minor comment:

Pg 8 line 13: why would CRAM be photochemically labile? Since CRAM does not likely absorb solar radiation, I would not expect it to be susceptible to direct photodegradation (perhaps indirect is possible however).

Open Access This Peer Review File is licensed under a Creative Commons Attribution 4.0 International License, which permits use, sharing, adaptation, distribution and reproduction in any medium or format, as long as you give appropriate credit to the original author(s) and the source, provide a link to the Creative Commons license, and indicate if changes were made.
In cases where reviewers are anonymous, credit should be given to 'Anonymous Referee' and the source.
The images or other third party material in this Peer Review File are included in the article's Creative Commons license, unless indicated otherwise in a credit line to the material. If material is not included in the article's Creative Commons license and your intended use is not permitted by statutory regulation or exceeds the permitted use, you will need to obtain permission directly from the copyright holder.
To view a copy of this license, visit https://creativecommons.org/licenses/by/4.0/

Response to Reviewer Comments – McKee et al. 2024

Please find below responses to reviewer comments (in blue) arising from our manuscript "Cycling of bioavailable carboxyl-rich alicyclic molecules and carbohydrates in Baffin Bay".

We greatly appreciate the constructive comments from the three reviewers which have substantially improved the manuscript. The principal concerns of the reviewers were largely methodological in nature, but also pertained to our interpretations of biological availability and scientific presentation of cycling mechanisms. To a lesser extent, reviewers requested the manuscript undergo better conformance to Nature Communications guidelines and some additional text be added.

We have gone to extensive lengths to address their concerns, extract matching SPE samples and generate a new NMR dataset comparison to validate the accuracy and robustness of our NMR method. To generate this new dataset, we incurred several major delays, including NMR instrument downtime/maintenance, the procurement, delivery and validation of new equipment needed for extracting and drying down SPE-DOM isolates and verifying their robustness. In the main manuscript and in the Supporting Information (SI), we have significantly bolstered the scientific discussion of cycling mechanisms, Baffin Bay trends and NMR methods details as requested. Overall, this work has significantly improved the total seawater NMR method and the science presented in the manuscript. We hope the reviewers and editor now agree that our manuscript represents a significant advance; one suitable for publication in Nature Communications. The manuscript (5308 words including figure captions and methods) and Supporting Information (SI) have been expanded. The SI now includes 2 new discussion sections, 5 figures, 4 tables.

Reviewer #1 (Remarks to the Author):

McKee et al. Cycling of bioavailable carboxyl-rich alicyclic molecules and carbohydrates in Baffin Bay

The complexity of chemical structures of dissolved organic matter (DOM) has limited our understanding of the exact pathways of carbon cycling in the ocean. McKee et al. used a new method to quantify the concentrations of total carbohydrates (considered to be labile) and CRAM (considered to be recalcitrant) using samples collected from the Baffin Bay in the Canadian Arctic. The authors observed degradation of the labile as well as recalcitrate components with depth in the Baffin Bay, challenging the traditional view that CRAM should be more persistently present in deeper water.

The value of this manuscript is the development of a novel method using total seawater DOM proton (1H) nuclear magnetic resonance (NMR) spectroscopy, which significantly enhanced the recovery of DOM, leading to the quantification of CRAM degradation in deeper water of up to 34% of its production in the surface ocean. This finding is profound and may change our view of CRAM as a refractory marker for DOM cycling in the ocean. However, I found the authors to sell themselves short in their claims, which raise questions that must be answered before people can adapt this method for improving our understanding of the mechanisms of DOM degradation in the ocean.

My first question is the comparison of the methods. The authors claimed that the traditional salt-free DOM extraction methods (ultrafiltration and/or solid-phase extraction) can only recover less than 20-40% of total DOM, which also induce chemical and isotopic biases that impede the characterization of DOM compositions. It is desirable that the authors perform these traditional analyses for comparison with the total seawater DOM proton (1H) NMR method, rather than relying on literature reports.

We have performed a direct comparison of contemporaneous total seawater DOM vs. SPE-DOM for n=4 Baffin Bay and the Canadian Arctic Archipelago samples. A comparison of our [TCHO] and [CRAM] concentrations between SPE-DOM and total seawater is revealing. Specifically, these new results in our updated Supplementary Information reveal that between 50-70% more CRAM and TCHO is present within total seawater spectra vs. SPE-DOM spectra. In addition, the ratio of CRAM:TCHO is nearly identical for watergate total seawater vs. SPE-DOM (n=4 average of-6±9% difference), suggesting that SPE DOM does not preferentially isolate CRAM over TCHO, but does miss a seemingly identical fraction of both compound classes in its recovery.

That leads to my second question: How would the DOM chemical structures differ between NMR, ultrafiltration and solid-phase extraction methods? This question should be answered by performing all three types of analyses using the same sets of samples. I did not find these analyses in the method section.

We believe ultrafiltration experiments fall beyond the scope of the present study. Moreover, these require specialized equipment and large sample volumes unavailable to us at the present time. Thus, we are unable to speak towards how high molecular weight (HMW) DOM chemical structures would specifically be different from contemporaneous SPE- or total- seawater samples in Baffin Bay. Ultimately, we feel this comparison is beyond the scope of our current study and should not be a requirement for its publication. However, we note the existing literature suggests significant broad differences. For example, exceedingly high TCHO are found within purified HMW DOM which is also a function of how these UF experiments are performed (concentration factors, membranes used, depths sampled). We know that SPE-DOM isolates far less TCHO and far more CRAM than HMW DOM in the open ocean[1,2]. We also know from the literature

that SPE-DOM isolates far fewer biomolecules (e.g. 2-3x less amino acids, etc.) than HMW DOM (e.g. Ianiri et al., 2022)[3].

My next question would be the actual mechanism for the degradation of CRAM in deeper water of the Baffin Bay. Although the authors use the word "bioavailable" in the title, implying microbial degradation of CRAM, no experimental data were provided. This question should be answered by performing cultivation experiments as in Zheng et al. (2022)[4] that was cited by this manuscript.

Microbial heterotrophic remineralization is the dominant mechanism through which we explain CRAM and TCHO cycling at depth, supported by previous studies. We agree that the use of "bioavailable" in the title could be perceived as problematic given, we did not perform incubation experiments. It also limits the possibility abiotic cycling mechanisms (e.g. photochemical oxidation). Following the reviewer's suggestion, we have changed the title to "*Cycling of labile and recalcitrant carboxyl-rich alicyclic molecules and carbohydrates in Baffin Bay*" which we feel is more appropriate given the exploration of "two-pool" CRAM and TCHO cycling in our dataset.

We agree that cultivation experiments represent an enticing future area of research, however, respectfully feel they are beyond the scope of the current study, represent considerable logistical constraints, and should not be a requirement for publication of this work. Much of what we have learned about DOM cycling is from fundamental concentration measurements in the ocean. A reasonable set of published mechanisms to infer processes that could be responsible for these trends is commonly used in this body of work. For example, existing DOM removal mechanisms (e.g. heterotrophic microbial respiration) has been inferred as the primary mechanism for global removal of deep ocean DOM as an explanation for changing deep ocean DOC concentration gradients (e.g. Hansell and Carlson, 2013)[5] without performing incubations. We have done our best to clarify CRAM production and removal mechanisms, specifically those evoked by Zheng et al., 2022[4], within the main manuscript text and we hope this satisfies the reviewers and editors.

Ultrafiltration experiments would similarly require additional ship time, water samples, equipment, students, and grants that we do not possess at the present time. We feel it is paramount that our results be made available to the scientific community as soon as possible and thus have decided not to delay by several years to perform these analyses. This represents an excellent future set of experiments we intend to apply for research funding to perform with a new set of PhD students and postdocs.

The authors may have a wonderful opportunity to test a hypothesis about CRAM degradation enhanced by the priming effect of more labile DOM (carbohydrates or proteins) accompanying CRAM going down the water column in the Baffin Bay. This would make the paper much stronger by telling a compelling story of the mechanisms of DOM degradation rather than a simple report of what happened (the decrease in CRAM abundance with depth).

Following other reviewers' comments, we have strengthened the discussion of possible cycling mechanisms (including photochemical oxidation, heterotrophy, priming, etc.) throughout the main text.

Priming is a difficult mechanism to explore without incubation experiments. The premise of priming is that some RDOC (or more total DOC) will be removed when higher levels of LDOC are present. A simple test of this idea would be to assume that higher intrinsic %TCHO or [TCHO] in the surface mixed layer would ultimately result in more [DOC] loss at depth or that with priming (and a larger depth specific DOC

gradient) higher %CRAM or [CRAM] at would also be observed at depth. From the plots below, we do not see evidence this is happening and have added a sentence to the main manuscript to reflect this (p. 8, L24). Relatively weak relationships are observed between station [DOC] depth loss and surface %TCHO or [TCHO] (Figure below).

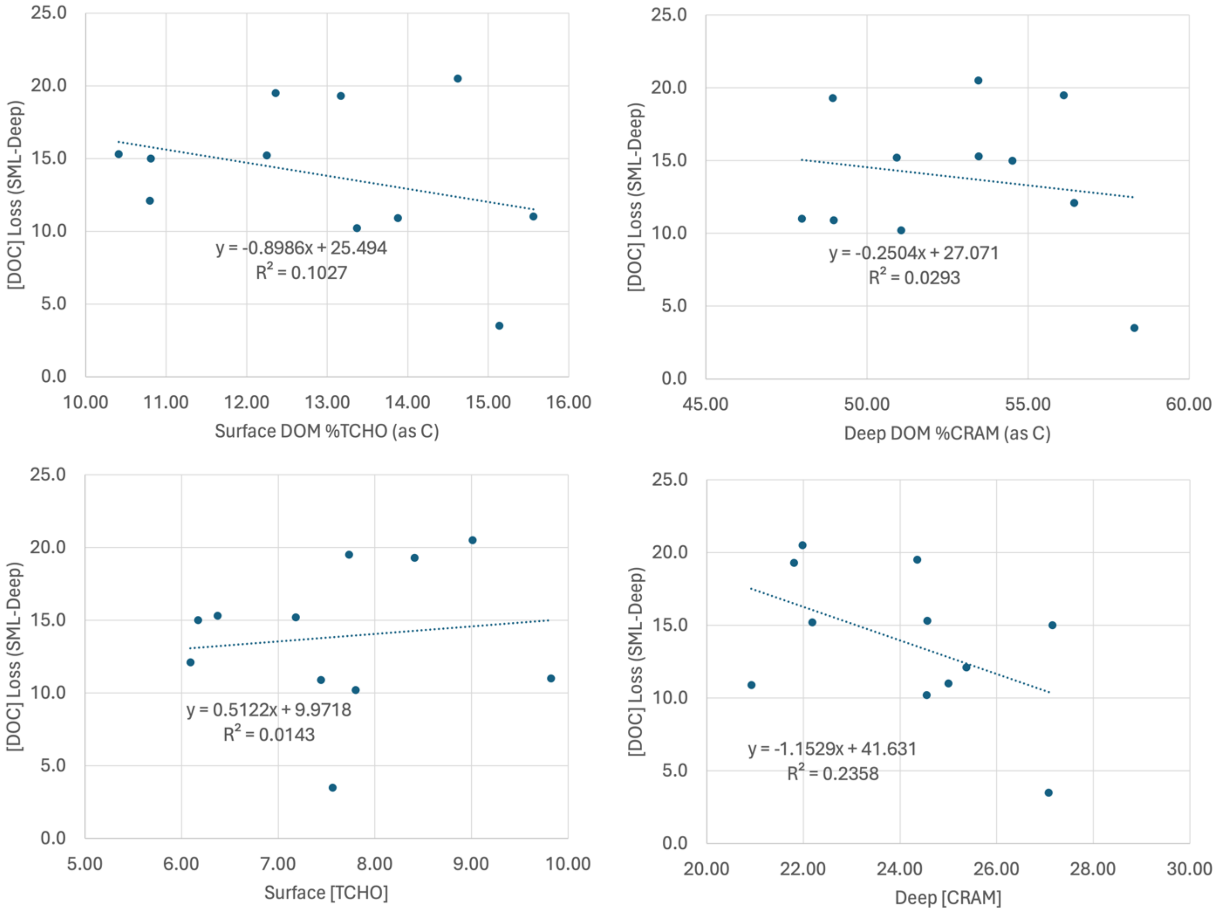

There is a minor correlation between DOC loss at depth with deep [CRAM] (Lower right panel, Figure above), which would not suggest "priming" as a major mechanism, so much as it would instead suggest that with more DOC cycling (loss) there is more CRAM made. This is the classical paradigm of deep CRAM cycling that has been established (e.g. CRAM builds up with slow microbial heterotrophic remineralization of organic matter). Additionally, a plot of surface [TCHO] and deep [CRAM] suggests increases in [CRAM] with depth are not primarily related to surface [TCHO] (Figure below).

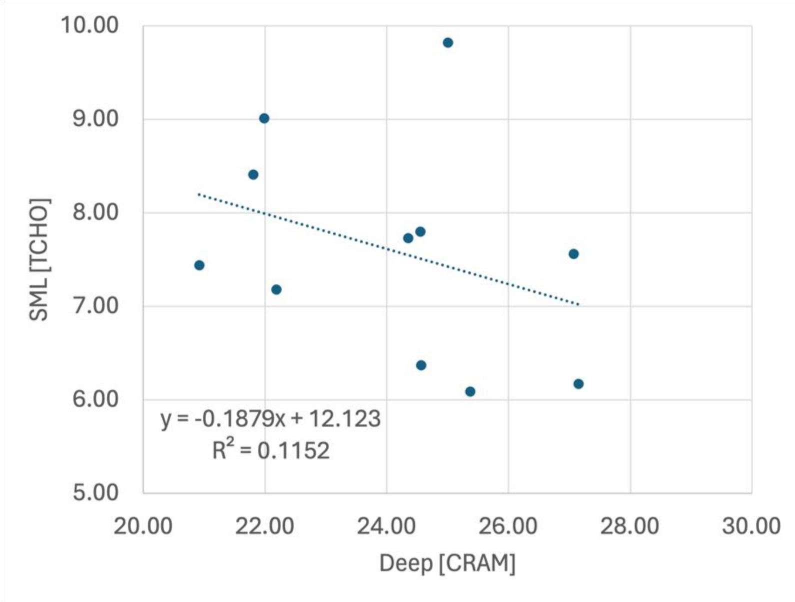

The authors should also provide more thorough information of the unique environment of the Baffin Bay: the terrestrial input of nutrients to the seawater in Baffin Bay, the impact of land-derived organic matter on the complexity of DOM in Baffin Bay, and the production of phytoplankton contributing to the fresh labile DOM in your samples.

This is a wonderful suggestion and one that has strengthened our manuscript. We have added a new paragraph in the main text about TCHO and CRAM concentration changes within the dominant current systems and water masses of Baffin Bay (new paragraph p. 7, L11-29). Unfortunately, we are now at the length limit for this article (5,000 words) with other reviewers requesting more methods details in the main article. Thus, additional hydrographic setting and terrestrial DOC discussion to the Supplementary Information section.

Other comments are line-by-line:

Line 42: "The Arctic Ocean.." should be a new paragraph. Change made.

Line 55: "It was inferred.." should be a new paragraph. – This statement is part of the previous topic thus we kept it as part of the same paragraph, however a new paragraph was started at "Subsequent ^{1}H-NMR…" to improve the structure of the writing.

Line 62: In "UDOM", what is U representing? – Changed to "HMW DOM" throughout. The 'U' represents "ultrafiltered" DOM.

Line 106: This paragraph should move up above line 100. – original manuscript line 100 discusses broadly what was presented in this manuscript, while line 106 provides specific data from our findings. For this reason, we left them in the original order to provide a logically progression of information.

Line 111: 100m, 200m: Should be "100 m, 200 m." The same correction is needed in many other places. Change made.

Line 115: Spell out "MBTH". – MBTH is "3-methyl-2-benzothiazoline hydrazone; a colourimetry measurements of sugar aldehydes", this has been added in parentheses here.

Lines 118-119: The sentence "This work is beyond.." is distractive. Suggest to delete. Change made.

Line 124: How does this "missing" CRAM differ in chemical structure from UF- or SPE-derived CRAM" This is a great comment and an area for a follow study. We cannot infer differences in CRAM chemical structure using only ^{1}H-NMR. One would need to use FTICR-MS, 2D NMR or similar techniques, unfortunately, this analysis cannot be performed on total seawater samples.

Line 143 and many other places: A space is needed before and after the "=" sign. – Change made.

Lines 161-162: Is it meaningful to report the p value as 0.0000028 or 0.000000041. Should the p value < than 0.001 be enough? – Change made.

Lines 178-179: "This CRAM is biologically available and rapidly removed in the surface ocean". It will be good to support this claim with experimental data. CRAM concentration gradients alone are sufficient for studies examining the relative abundance and cycling of DOM. The change in abundance of DOC and DON from the surface to deep ocean has similarly been evoked to describe biological availability. Incubation experiments would require additional water samples that we do not posses and time to establish this method (equipment, expertise, etc.) in our facilities. We feel it is paramount that our results be made available to the scientific community as soon as possible and thus have decided not to delay by several years to perform these analyses. This would, however, be an excellent experiment to be run within a newly funded research grant.

Lines 213-215: The statement here is too speculative. We have reframed this sentence as a hypothesis: "Surface to deep gradients in CRAM occur largely in concert with changes in DOC concentrations. We hypothesize a large portion of the marine DOM cycle is mediated through both autotrophic production and heterotrophic cycling of this population of chemically-distinct molecules."

Lines 220-222: The statement of 14C age is too much speculation. – A speculative implications statement is justified here and is presented as a testable hypothesis for the community to follow-up on. We have updated text to "*While significant loss of bioavailable CRAM and TCHO is observed, we also hypothesize the long residence time of Baffin Bay Deep Water (360-690 years)*[34] *will result in long-term storage of recalcitrant CRAM and TCHO in deep Baffin Bay. If true, we hypothesize that deep CRAM and TCHO populations should have greater average* ^{14}*C-ages, and potentially molecular diversity, resulting from slow microbial heterotrophic degradation imparting recalcitrance before entering meridional overturning circulation in the North Atlantic. This hypothesis will be readily testable with future development of CRAM isolation techniques.*"

Figures 1B and 1C: The temperature and salinity impact on DOM composition between stations on either side of the Baffin Bay should be discussed. We find no significant average %composition difference between the transects on either side of Baffin Bay. This is presented in the main text "*a one-way ANOVA resulted in no significant average difference between the two transects for surface %TCHO (F(1, 38) = 2.87, = 0.10), deep %TCHO (F(1, 41) = 0.98, = 0.33), surface %CRAM (F(1, 38) = 0.31, p = 0.58) or deep %CRAM (F(1, 41) = 1.93, p = 0.17).*". The cycling of DOC and DON will be the focus of a follow-up paper we are hoping to submit this year.

Line 251 and other places: 50um should have a space in between. – Change made.

Line 452 and other places: 2mL should have a space in between. – Change made.

Line 447 and other places: 4.8ppm should have a space in between. – Change made.

In summary, I like the way the paper is trying to accomplish, but the story needs to be beefed up tremendously. I would very much like the story to be fully developed taking consideration of my comments and suggestions. At this stage, it does not meet the requirement for publication in NC.

We greatly appreciate Reviewer 1's comments which, through addressing, have significantly improved the manuscript. However, we respectfully disagree that our study, which presents the first CRAM concentrations for any aquatic system and demonstrates a new CRAM cycling paradigm, does not meet the requirements for publication at *Nature Communications*.

We have made significant efforts to bolster the CRAM and CHO cycling story and include discussion of mechanisms specified by the reviewer. We have gone through great experimental lengths to perform a new set of DOM extractions, NMR analyses and direct comparison of SPE vs. total seawater DOM at the request of reviewers. A few of the reviewer's requested changes fall beyond the scope of the current manuscript but are insightful future research directions to be carried out. For example, a request to return to Baffin Bay, perform new ultrafiltration experiments and microbial incubation experiments is not feasible and should not be required for publication of this manuscript. Through implementing the majority of their requested changes, and those of Reviewer-2 and Reviewer-3, we hope Reviewer-1 now agrees our manuscript meets the requirements for publication in NC.

Reviewer #2 (Remarks to the Author):

Dear Editor,

Sub: Review of a manuscript (NCOMMS-23-27002-T) entitled "Cycling of bioavailable carboxyl-rich alicyclic molecules and carbohydrates in Baffin Bay" by McKee et al.

While the study presents interesting findings related to the cycling and estimation of the quantities of two major structural groups of dissolved organic matter (DOM) - total carbohydrates and carboxyl-rich alicyclic molecules (CRAM) in Baffin Bay - and proposes a two-pool CRAM model, I regret to inform you that the manuscript, in its current form, falls short of the standards expected for publication in Nature Communications. The following concerns underlie my recommendation:

The authors have employed a previously published total seawater 1D 1H NMR technique to study the cycling of DOM, which is advantageous for its comprehensive analysis without the need for a preconcentration step and avoidance of chemical or size-based extraction biases. However, the well-known limitations of poor resolution and peak attenuation around the water signal raise questions about the accuracy of the quantitative analysis of DOM structural groups, such as total carbohydrates and CRAM. Consequently, I recommend that the authors validate their findings using complementary analytical approaches, such as high-resolution mass spectrometry.

We appreciate the constructive comments from the reviewer. We acknowledge the effect of the water suppression technique on potentially suppressing carbohydrate anomeric protons (between 4.5-5.5 ppm), which could lead to an underestimation of the carbohydrate contribution, as described by Lam et al. (2007)[7] and Fox et al. (2018)[8]. However, we believe this effect will be minimal for several reasons:

- The suppression effect will be consistent across all samples, which is crucial for a comparative study like ours.
- The carbohydrate anomeric proton is not the major hydrogen bond of the carbohydrate, representing less than 20% of the total carbohydrate ^{1}H-NMR region.
- We can still quantify the other major ^{1}H-NMR bands of carbohydrates, and by using the correct H:C ratio, we can quantify the majority of carbohydrates.
- The effect of water suppression on CRAM (Carboxyl-Rich Alicyclic Molecules) will be negligible since the CRAM region was integrated from 0.2-3.2 ppm, which is lower than the water suppression region of 4.5-5.5 ppm.

To verify the effect of water suppression and address issues such as attenuation around the water peak and its impact on accuracy, we extracted four water samples from Baffin Bay using PPL-SPE, then redissolved and analyzed them using both water suppression and traditional D_2O techniques. We compared these results to the H-NMR of total seawater (see *Supplementary Information*). Our new results suggest that the water suppression method yield substantially higher TCHO and CRAM concentrations than traditional D_2O NMR measurements.

Due to ion suppression, matrix effects and varieties of ionization efficiency between DOM compounds, high-resolution mass spectrometry is not quantitative and cannot produce CRAM concentrations, making it unsuitable for assessing our data quality or invalidating our results. Additionally, the current techniques of FTICR-MS are not capable of ionizing and detecting carbohydrates efficiently.

While this study demonstrates that not all CRAM is recalcitrant, but a fraction of the CRAM pool of DOM is biologically labile, it is essential to reinforce these conclusions through robust evidence. I urge the authors to undertake additional experiments or analyses to confirm and strengthen the proposed two-pool CRAM model, ensuring the reliability of their results.

As per comments from other reviewers, additional NMR experiments have been performed and demonstrate our method is far better at quantifying total [CRAM] concentrations than existing NMR and SPE DOM isolation methods. However, additional research cruises and microbial incubations and measurements are future research directions that fall beyond the scope of the current study. The CRAM concentrations reported here provide the basis for such future work much as the first high resolution ocean DOC concentrations provided the basis for more detailed incubation and chemical analysis studies. Seminal work by Dr. Hansell and others have revealed geographic areas important to DOM cycling through the use of DOC concentration measurements alone (e.g. Hansell and Carlson, 2013)[5]. Our study provides a similar scientific basis through which additional targeted studies and more detailed information on CRAM cycling can be funded and carried out in the future.

Specific comments and questions:

1. 1D 1H NMR suffers from poor spectral resolution due to the spread of H-H J-coupling multiplets over a limited chemical shift range of ~10 ppm, particularly for highly intricate molecular mixtures such as DOM. Additionally, the 600 MHz NMR employed in this study is not an optimal instrument for resolving the signals from millions of chemically distinct compounds present in DOM and improving the detection of signals from very dilute compounds. The 1H NMR integration region of CRAM (3-0.5 ppm) chosen in this study may have a significant contribution from the protons of other structural groups of DOM due to overlapping. How did the authors account for this bottleneck? How confident are the authors about quantifying exclusively the CRAM fraction of DOM in the highly crowded 1H NMR spectra?

As the reviewer points out, the complexity of DOM, which consists of tens of thousands of individual compounds, results in poor spectral resolution compared to traditional pure compound ^{1}H-NMR spectra. Many DOM compound classes share similar backbone structures with slight variations, leading to small chemical shifts within these classes. The combination of chemical shifts and intensities of these thousands of compounds results in broad, unresolved peaks. The J-J H coupling has a minor effect in complex mixtures like DOM. In previous experiments, we tried disabling the J-J coupling in ^{1}H-NMR DOM spectra, but we did not observe an improvement in spectral resolution.

We respectfully disagree with the reviewer; we believe that using a 600 MHz NMR with a 5 mm inverse H-C/N-D cryoprobe is ideal for increasing the sensitivity and S/N ratio for the quantitative aspects of this study. The use of a cryoprobe on a 600 MHz NMR can significantly enhance sensitivity due to reduced thermal noise, often making it comparable to or even better than a non-cryoprobe 900 MHz NMR. The main objective of this study is to quantify different compound classes (e.g., CRAM), not to identify the chemical structures of individual compounds. If our aim were to identify the chemical structures of individual compounds, we agree that using a 900 MHz NMR for higher spectral resolution would be appropriate, but this is not the aim of this study.

Regarding the contribution from the protons of other structural groups of DOM due to overlapping, we have taken that into account. This is why we use 2D-correlation and PCA loading to identify the specific band-associated compound classes and determine the exact integration regions for these compounds, as

explained in our previous work (e.g., Fox et al. 2018, Abdulla et al. 2010, 2013)[8–10]. Based on our previous ^{1}H-NMR studies in different ecosystems, we are very confident in our integration approach to quantify the different compound classes.

2. To confirm the 1D 1H NMR-based findings of this manuscript, it would be beneficial to have complementary evidence, such as ultrahigh-resolution FT-ICR mass spectrometry analysis. Unfortunately, 2D NMR analysis is not feasible due to the extremely low concentration of the samples.

We agree with the reviewer that 2D NMR techniques, such as ^{1}H-^{1}H COSY and TOCSY, would be valuable for confirming the structure. However, these methods are not feasible due to the low DOC concentration. Additionally, as the reviewer is aware, 2D NMR techniques are not quantitative, necessitating the use of 1D-^{1}H-NMR for quantification.

FT-ICR-MS is not suitable for use on total seawater. Moreover, it is non-quantitative for complex organic matrices for reason mentioned above (ion suppression, matrix effects and varieties of ionization efficiency between DOM compounds) making it unsuitable for assessing our data quality or invalidating our results.

3. How distinct are the two CRAM fractions (recalcitrant vs. labile) of DOM structurally? What factors make a specific fraction of CRAM biodegradable? Is the labile CRAM fraction only biodegradable, or is it also photodegradable?

This is an excellent area for future work distinct CRAM populations. ^{1}H-NMR in and of itself is unable to differentiate between "types" of CRAM. Thus, our two-pool model is inferred from [CRAM] concentrations alone. We hypothesize that labile CRAM could be biologically bioavailable and photochemically degradable. We have added this latter mechanism to the main article text where possible. We have also re-titled the manuscript to be more inclusive of abiotic CRAM and TCHO cycling mechanisms.

4. When using the 1H NMR pulse sequence reported by Lam et al. (Analyst, 2008, 133, 263"269), signals up to 1.1 ppm on each side of the water signal (in the range approximately 5.9-3.7 ppm, considering the water signal appeared at 4.8 ppm) will be attenuated, which drastically affects the quantification of carbohydrates. The authors should clarify how accurate their quantification of total carbohydrates is under these conditions.

We provide a detailed SPE comparison and analysis of the attenuation and quantification of TCHO in this response and in the Supplementary Information section. A direct comparison of SPE vs. total seawater DOM measured via water suppression resulted in 52-70% higher TCHO concentrations than SPE-DOM. The comparison also revealed the watergate method is more sensitive than traditional D_2O NMR. Our SPE D_2O experiment [TCHO] values were far more strongly affected by residual water and attenuation than that of watergate. Whereas total seawater and SPE watergate experiments had a similar CRAM:TCHO ratio, SPE D_2O had a far higher ratio due to this attenuation and lower quantification of TCHO. This despite our significant efforts to fully deuterate, sonicate, centrivap (-85°C) SPE-DOM to dryness repeatedly to eliminate H_2O in the sample.

5. Figures: There are only two figures in the main text, one of which (Figure 1) is adapted from an already published paper. Since this is an original research article and not a review, the adapted figure does not add any new information to strengthen the manuscript. I understand that it is important to show the location of the sampling sites, so the authors should consider providing this figure in the Supplementary Information and using the space in the main text more effectively.

We appreciate this comment. Figure 1 is adapted from an earlier paper and provides critical oceanographic context for Baffin Bay and its current systems – without which, the reader would have difficulty understanding the dominant currents systems, water mass origins, and the DOM transects as presented. Given other reviewers' requests for additional hydrographic discussion, keeping this figure is critical. As per Nature's guide to authors, we are allowed 5000 words and up to 10 figures. That said, if the editors feel we should move the figure, we'd be willing to do so upon acceptance.

Additionally, it is challenging to read the scale in Figure 2; therefore, the resolution and font size should be increased for better visibility. Changes made.

6. The findings of this manuscript are mainly based on the 1D 1H NMR of total seawater; however, the 1H NMR spectra are not shown in the manuscript, not even in the Supplementary Information. To evaluate the authors conclusions, a comparison of 1H NMR spectra should be provided as a figure, at least in the Supplementary Information.

An example of our 1D spectra is shown in the SI (Figure S3). We have included new NMR spectra that compare the Total seawater H-NMR with SPE-DOM from the same sample using two different NMR methods (watergate vs. D2O; Figure S4).

7. Lengthy abstract: The abstract contains approximately 273 words, which is much longer than the 150-word limit recommended by the journal.

We have reduced the abstract to <200 words as per the guide to authors (https://www.nature.com/ncomms/submit/article). This and the following comments below stemmed from the invitation and transfer of the article directly from Nature Geoscience.

8. Lines 15-26: Literature should not be cited in the abstract.

We have removed citations from the abstract.

9. Lines 22-24: The statement regarding the solid-phase extraction efficiency (<20-40%) is not correct. The solid-phase (PPL) extraction efficiency for DOM has been greatly improved over the past decade, and the recovery rate for both deep and surface ocean bulk DOM was 61%. (Reference: Green et al., Marine Chemistry, 2014, 161, 14-19; An intercomparison of three methods for the large-scale isolation of oceanic dissolved organic matter).

We have expanded the range of extraction efficiency in the sentence to more accurately reflect commonly reported UDOM (10-30%) and SPE recoveries (40-60%) in this sentence. How SPE is performed matters and we have found that most SPE studies fall short on these details. Green et al. (2014), is an exception to this of course. But not all SPE-DOM studies use the method by Green et al., 2014[11] and still rely on the older methods[12]. SPE efficiency is a function of sample salinity, resin loading, water volume, DOM concentrations, molecular diversity and eluent volume. We still commonly see reported extraction efficiencies of "40%" where %yields were not measured, but simply inferred from the early Dittmar L&O paper[12]. In our SPE vs. total seawater comparison, we observed SPE efficiency between 39- 55%DOC.

10. Line 75: It is "mass spectrometry" but not "mass spectroscopy".

Change made.

11. Lines 75-76: The abbreviation of CRAM is "carboxyl-rich alicyclic molecules," not "carboxyl-rich alicyclic material."

Change made.

12. Line 116: Please replace "From our integrations" with “Based on 1H NMR integrations”.

Change made.

13. Line 126: The statement regarding the SPE-DOC recovery (~40%) is not correct. Please refer to comment 9.

We have changed this to “40-61%” and cited Green et al 2014[11] here and update relative CRAM contributions to UDOM and SPE DOM in the following sentences.

14. Line 212: SPE-DOM is not a technique, but it is the fraction of DOM extracted by the SPE technique. – Updated to “SPE technique” rather than “SPE DOM technique”

15. Line 257: Please replace “was” with “were” Change made.

16. Lines 262 and 500: “dH” denotes the enthalpy change in thermodynamics. The chemical shift is denoted by the symbol "??" but not "d". Please replace "dH" with "??H" to denote proton chemical shift and ensure that it is corrected throughout the main text and supplementary information, wherever applicable. Change made.

17. Line 474: Please replace “was” with “were” Change made.

18. Line 475: TMS (tetramethyl silane) is a good NMR internal standard for most organic solvents, but it has very low solubility in water and is not generally used as an internal reference in aqueous solutions. Since all the spectra were recorded in water in this study, the authors should clarify the use of TMS as an NMR internal standard instead of DSS (sodium 4,4-dimethyl-4-silapentane-1-sulfonate) or DSA (4,4-dimethyl-4-silapentane-1-ammonium trifluoroacetate) which are preferred NMR standards for water samples. Did the authors intentionally add TMS to the NMR sample, or did they mistakenly assume naturally occurring silicate species as TMS?

The low solubility of TMS works in our favor, as it prevents overwhelming the ^{1}H-NMR signal of the low DOC in the samples. We avoided using DSS because it would dominate the NMR signal, particularly the DOM signal below 1 ppm during the long NMR acquisition experiment. Instead, we rely on the trace amount of TMS contained in the D_2O, which was added to the seawater samples during preparation for locking and shimming of the NMR.

19. “Results and Discussion” section heading is missing in the main text. Change made.

20. The authors should provide a complete description of 1H NMR experimental and processing parameters at least in the Supplementary Information to ensure reproducibility.

Change made. We have merged our two methods sections and added the relevant NMR experimental and processing parameters in the main text.

21. Data availability statement: The data availability was not indicated. It is necessary to deposit time-domain and processed NMR data that support the findings of this manuscript to repositories to ensure reproducibility.

We have updated our data availability statement. Prior to acceptance, we will provide processed, phased and baseline corrected NMR spectra (0-5ppm; n=100) in the Supplementary Information and within Open Science Framework via a static DOI with CC0 1.0 Universal access conditions.

Reviewer #3 (Remarks to the Author):

McKee et al. present an interesting study that suggests a "labile" CRAM pool in the surface ocean, contrary to its typical classification as a major component of the "refractory" DOM pool. The concept of 1H NMR analysis on whole water samples is also highly interesting, and results of this study highlight its potential to re-evaluate our understanding of marine DOM composition beyond what we have learned from analyses of SPE-DOM. That said, I would have appreciated more details on the methodology, particularly in the calculation of carbon-based concentrations from 1H NMR data. Perhaps this could be supplied in the SI, with PCA analyses.

We appreciate the reviewer's recognition of the novelty of our study. In response, we have consolidated our online and in text methods sections which now provide more details on the methodology and equations used to calculate the carbon-based concentration from ^{1}H-NMR data.

I also wonder how these results compare to "% CRAM" results determined from 13C NMR (or is it 2D NMR?). e.g., Lines 52-55 state, "Through 13 C-NMR measurements it was estimated that ~50% of surface ultrafiltered high molecular weight DOM (HMW DOM; >1000 Da) was carbohydrates whereas both surface and deep HMW DOM were composed of primarily aliphatic molecules and carboxylic acids14,15."

Moreover, lines 72-72 reiterate "Based off 13 C-NMR, this material is found throughout the water column, but in higher relative total abundance in deep UDOM, suggesting a refractory "humic" background DOM component that lasts several oceanic mixing cycles15,21." I imagine there is a carbon limitation here, so only 1H NMR is possible without preconcentrating DOM, but I wonder if there are large differences between calculated %CRAM using this approach and that from Hertkorn et al. 2013.

As the reviewer knows, due to the low abundance of ^{13}C and its low gyromagnetic ratio, the sensitivity of ^{13}C is approximately 1.72 x 10^{-4} relative to that of ^{1}H. This makes it nearly impossible to analyze ^{13}C-NMR on natural marine DOC samples. Additionally, as the reviewer mentioned, the Hertkorn et al. 2013[2] study is based on an isolated fraction of DOM (UDOM) with a total recovery of 20-30% of marine DOM. This makes it difficult to quantitatively compare our % CRAM obtained using total seawater ^{1}H-NMR with their results.

However, we performed an additional ^{1}H-NMR comparison between SPE-PPL and total seawater for four Baffin Bay samples. These results indicate that SPE-PPL underestimates the CRAM concentration by a factor of 2-3 times compared to our ^{1}H-NMR data (Table S4). Given that SPE-PPL is more efficient at extracting CRAM-like compounds (humic background) relative to UDOM, it can be inferred that UDOM also underestimates the CRAM concentration.

I also wonder if Hertkorn et al. (2013) suggest 51% (5 m) - 56% (5446 m) of SPE-DOM as CRAM, wouldn't the same trend be observed as this study, if DOC extraction efficiencies do not change with depth (as suggested by Ksionzet et al. 2016, Science(354), doi: 10.1126/science.aaf7796)? There will be more SPE-DOC in the surface than at depth, and 51% vs. 56% CRAM would mean higher CRAM concentrations in the surface? All this is to say that these additional details/answers to these questions may help explain why the results obtained here are unique and why they suggest that CRAM in the surface exists in at least 2 pools (labile and refractory). Additional details should also help explain why the %TCHO and %CRAM calculations are so noisy.

Since we do not have SPE-DOM ^{1}H-NMR throughout Baffin Bay, we cannot confirm SPE-CRAM distributions would follow the depth trend of Hertkorn et al., (2013)[2]. However, through our new SPE-DOM vs. total seawater DOM comparison, we do find consistent results with Hertkorn et al., (2013)[2] with CRAM comprising between 50-53% of SPE-DOM, albeit we did not observe more %CRAM in our one deep SPE sample (Station 210, 800m = 51.8% CRAM). Given the paucity of SPE-DOM %CRAM data, and a known chemical preference of the PPL resin, it is equally plausible the SPE isolates a similar CRAM population (and concentrations) throughout the water column, which would be more consistent with recent LMW-SPE DOM isotopic and NMR studies[13,14]. This would be wonderful to explore but requires work beyond the scope of our current manuscript.

We do not observe ubiquitous SPE extraction efficiency with respect to DOC as reported in Ksionzet et al.[15], (2016). Our SPE recoveries ranged from 39-55% DOC (Table S4), as R2 points out SPE recoveries can be as high as 61% (Green et al., 2014)[11]. SPE recoveries are quiet sensitive to a variety of parameters: resin loading (vol vs. resin weight used), resident DOM polarity, DOM molecular diversity (active site competition), seawater salinity (more active site competition) and eluent volume (e.g. Lewis et al., 2020)[16].

All this said, our SPE-DOM comparison work (Table S4, Figure S4) adds a few details that might help the reviewers' comments here. The core results of our SPE comparison study suggest that SPE isolates have similar %CRAM and %TCHO values, but also that by concentration, SPE isolates between 50-60% less total CRAM and 50-70% less total TCHO than is present in total seawater. CRAM:TCHO ratios are similar for both total seawater and SPE-DOM, suggesting no inherent preference for CRAM over TCHO. Instead, it appears SPE "misses" a similar population of TCHO and CRAM in all extractions that is simply not retained by PPL. Additionally, our watergate experiments on both sample types suggest improved quantification of TCHO vs. traditional D_2O ^{1}H-NMR. Even though we know a major CHO region is attenuated by water suppression, it is far less attenuation than was induced by a residual water peak in our traditional SPE-DOM D_2O ^{1}H-NMR measurements. We have added discussion of this sample comparison in the Supplementary Information.

Another thought: if this work suggests that CRAM is not as refractory as previously suggested, couldn't this work also imply that TCHO is not as labile as previously thought?

We can't definitely state that TCHO is not labile, as it could be potentially going through internal cycling within the DOM pool, as a specific TCHO compounds in the surface could be different structure than the TCHO compound in deep ocean but the quantity could similar. There are lacking information about the individual TCHO compounds as most of the current study depend on hydrolysable carbohydrate or detecting free monosaccharide which account for small % of the TCHO and didn't have the structure or molecular formulas of individual TCHO compounds. That said, previous studies have reported similar vertical distributions of mono- and polysaccharides (e.g. Pakulski and Benner 1994)[17]. They find polysaccharides as an active component of the upper ocean carbon cycle, but also find 5-10µM TCHO at depth. This fraction was dominated by both hydrolysis-resistant polysaccharides (e.g. chitin and cellulose) and mono-saccharides that are less labile at depth. We have added a some of this text to p. 7 L7 of the manuscript. Following this and comments from other reviewers, we have revised our title from "bioavailable" to more accurately represent our discussion of "Labile and recalcitrant" CRAM and TCHO.

Minor comments listed below:

Line 115: what is MBTH? – MBTH is "3-methyl-2-benzothiazoline hydrazone; a colourimetry measurements of sugar aldehydes", this has been added in parentheses.

Lines 157-159: Are these average values that are reported, like those reported for %TCHO and %CRAM? What is the standard deviation? It does seem like there is a lot of variability (Figure S2), so perhaps could help in understand how these values are significantly different (apart from the ANOVA that is discussed next).

Yes, these are average values as indicated at the start of the sentence. We have added standard deviations to the text. However, we decided against adding error bars to Figure S2 as the figure would become cluttered and difficult to read.

Lines 161-163: I would argue that a decrease in 1 umol/kg by NMR vs 3 umol/kg by MBTH is not consistent, but rather a large difference, especially if the decrease is from 7 to 6 umol/kg (NMR) vs 7 to 4 umol/kg (presumably by MBTH).

The two methods and TCHO concentration gradients likely have overlapping errors (2-sigma). However, following these comments we have modified the statement to read: "*Previous work in the North Atlantic using the MBTH method observed a 3.0 µmol C kg^{-1} loss of labile TCHO with depth and the persistence of 5-10 µmol C kg^{-1} recalcitrant TCHO at depth which was dominated by both hydrolysis-resistant polysaccharides (e.g. chitin and cellulose) and mono-saccharides*[35]*. To first order, we observe a similar amount of labile vs. recalcitrant TCHO within the upper water column and deep waters of Baffin Bay, respectively.*"

Figure 2: the scales on the colour bars could be larger, it is hard to tell if West Greenland Current data and Baffin Island Current data are plotted on the same scale. – Changes made. Also, font sizes have been increased.

Figure S2: the labels are quite small and difficult to read. Any error associated with these points? I would also appreciate seeing the DOC data plotted in this manner (not just ODV plots). Changes made. We decided against adding error bars to Figure S2 as it would become cluttered and difficult to read. Instead, standard deviation values have been added in the main text.

Table S2 could also benefit from larger font. Change made.

Line 250: Is centrifugation necessary with filtered samples? – Yes, the ^{1}H-NMR technique is highly sensitive to residual POM. Centrifugation is least invasive way to remove POM without contaminating the sample (e.g. with nylon, PES, PTFE from syringe filters).

Line 432: define KHP. – Updated to "potassium hydrogen phthalate (KHP)"

Literature Cited in this Response to Reviewers:

1. Hertkorn, N. *et al.* Characterization of a major refractory component of marine dissolved organic matter. *Geochim Cosmochim Ac* **70**, 2990–3010 (2006).

2. Hertkorn, N., Harir, M., Koch, B. P., Michalke, B. & Schmitt-Kopplin, P. High-field NMR spectroscopy and FTICR mass spectrometry: powerful discovery tools for the molecular level characterization of marine dissolved organic matter. *Biogeosciences* **10**, 1583–1624 (2013).

3. Ianiri, H. L., Shen, Y., Broek, T. A. B. & McCarthy, M. D. Bacterial sources and cycling dynamics of amino acids in high and low molecular weight dissolved organic nitrogen in the ocean. *Mar. Chem.* **241**, 104104 (2022).

4. Zheng, X. *et al.* Experimental Insight into the Enigmatic Persistence of Marine Refractory Dissolved Organic Matter. *Environ Sci Technol* **56**, 17420–17429 (2022).

5. Hansell, D. A. & Carlson, C. A. Localized refractory dissolved organic carbon sinks in the deep ocean. *Glob. Biogeochem. Cycles* **27**, 705–710 (2013).

6. Zeidan, S. *et al.* Using Radiocarbon Measurements of Dissolved Inorganic Carbon to Determine a Revised Residence Time for Deep Baffin Bay. *Frontiers Mar Sci* **9**, 845536 (2022).

7. Lam, B. & Simpson, A. J. Direct 1H NMR spectroscopy of dissolved organic matter in natural waters. *Analyst* **133**, 263–269 (2007).

8. Fox, C. A., Abdulla, H. A., Burdige, D. J., Lewicki, J. P. & Komada, T. Composition of Dissolved Organic Matter in Pore Waters of Anoxic Marine Sediments Analyzed by 1H Nuclear Magnetic Resonance Spectroscopy. *Frontiers Mar Sci* **5**, 172 (2018).

9. Abdulla, H. A. N., Minor, E. C., Dias, R. F. & Hatcher, P. G. Transformations of the chemical compositions of high molecular weight DOM along a salinity transect: Using two dimensional correlation spectroscopy and principal component analysis approaches. *Geochim. Cosmochim. Acta* **118**, 231–246 (2013).

10. Abdulla, H. A. N., Minor, E. C. & Hatcher, P. G. Using Two-Dimensional Correlations of 13C NMR and FTIR To Investigate Changes in the Chemical Composition of Dissolved Organic Matter along an Estuarine Transect. *Environ. Sci. Technol.* **44**, 8044–8049 (2010).

11. Green, N. W. *et al.* An intercomparison of three methods for the large-scale isolation of oceanic dissolved organic matter. *Mar. Chem.* **161**, 14–19 (2014).

12. Dittmar, T., Koch, B., Hertkorn, N. & Kattner, G. A simple and efficient method for the solid-phase extraction of dissolved organic matter (SPE-DOM) from seawater. *Limnol. Oceanogr.: Methods* **6**, 230–235 (2008).

13. Broek, T. A. B., Walker, B. D., Guilderson, T. P. & McCarthy, M. D. Coupled ultrafiltration and solid phase extraction approach for the targeted study of semi-labile high molecular weight and refractory low molecular weight dissolved organic matter. *Mar Chem* **194**, 146–157 (2017).

14. Broek, T. A. B. *et al.* Low Molecular Weight Dissolved Organic Carbon: Aging, Compositional Changes, and Selective Utilization During Global Ocean Circulation. *Global Biogeochem Cy* **34**, (2020).

15. Ksionzek, K. B. *et al.* Dissolved organic sulfur in the ocean: Biogeochemistry of a petagram inventory. *Science* **354**, 456–459 (2016).

16. Lewis, C. B., Walker, B. D. & Druffel, E. R. M. Isotopic and optical heterogeneity of solid phase extracted marine dissolved organic carbon. *Mar. Chem.* **219**, 103752 (2020).

17. Pakulski, D. & Benner, R. Abundance and Distribution of Carbohydrates in the Ocean. *American Society of Limnology and Oceanography Inc.* **39**, 930–940 (1994).

Response to Reviewer Comments – McKee et al. 2024

We would like to thank the reviewers for their constructive and detailed comments. We have addressed the reviewers' comments and provide our responses (in blue) below, including information about the conversion of carbon content.

REVIEWER COMMENTS

Reviewer #1 (Remarks to the Author):

It is obvious that the authors tried hard to address my previous comments, with some answers satisfying. But my key concern or request for ground truth was not satisfied. Particularly these two questions:

We appreciate the reviewer's careful reading and constructive comments on the manuscript. We have addressed all the reviewer comments and questions in detail below.

1. The underlying mechanism allowing the total DOM proton (1H) NMR technical to be superior to ultrafiltration or SPE, which were claimed to induce chemical-, size- and isotopic-bias. Will the novel method remove these biases?

In short, yes. As we mentioned in the manuscript, the main advantage of this study is eliminating the biases caused by chemical- or size-fractionation of DOM in quantifying its major components. This method provides a more unbiased chemical structure characterization of the entire DOM, as it does not require any additional sample treatment like acidification, solvent extraction, ultrafiltration or drying.

2. It is thus contradicting to state that "it is plausible SPE isolates a similar CRAM population (and concentrations)" while the new method covers greater abundance of CRAM with the same chemical structures.

We can understand the confusion and apparent contradiction between these two sentences. As it is well known, SPE extraction techniques have a preferential (biased) chemical isolation of specific compounds based on the type of sorbent and elution solvent used. However, even under optimized conditions for specific compounds, SPE does not achieve 100% recovery. Recoveries typically range from 70% to 90% for most targeted analytes. For example, consider the extraction of pesticides and drugs from complex mixtures; these studies always conduct compound-specific recovery tests and adjust results according to the recovery percentage for each specific compound. In the case of CRAM, even though PPL-SPE may partially select for CRAM, it cannot recover 100% of each individual compound, which introduces another layer of bias when using SPE for quantifying CRAM. By using whole ^{1}H-NMR, we eliminate that extraction bias, allowing for more accurate quantification of the entire CRAM pool, even though the structures might be the same as those extracted by SPE.

Based on Figure S5 and Table S2, would it be possible to do a linear discriminant analysis (LDA) or similar comparison analysis to show what chemical compounds are distinctive or

as the authors claimed the novel method recovers CRAM populations having the same composition as the SPE.

We plan to address in a future study. In the current study, we only have four samples for which we conducted both SPE and Whole ^{1}H-NMR analyses. While this is sufficient to highlight the differences in chemical composition, as shown in Figure S1, it is not statistically sufficient to perform Linear Discriminant Analysis (LDA) or other chemical statistical analyses to explore deeper into the biases caused by SPE. The main aim of this study is to quantify the major compound classes (e.g., CRAM and TCHO) without focusing on identifying the changes in chemical composition between the SPE and whole ^{1}H-NMR methods.

Overall, there are more questions arising than what the revised paper can answer.
This work represents the beginning of a renewed understanding of the marine CRAM cycle. We have addressed all reviewer concerns, however, are planning several follow-up studies to answer those science questions arising that are beyond the scope of the current study.

There are many typos in the main text and supplemental information the authors need to correct, which are highlighted in their PDF documents I submitted.

We appreciate the reviewer's effort in highlighting these typos. We have corrected all of them in the revised manuscript. We detail these below referencing line numbers in the reviewer supplied .pdf files:

<u>Main Text:</u>
L36: 4,000-6,000 years
L40: Kept original text. DOM cycling without molecular transformation is possible (e.g. respiration of simple molecules to CO_2 vs. modification of complex macromolecules)
L72-74: Kept original text. Required change not specified
L101: Kept original text. Because many forms of CHO are detectable by NMR, and we integrate all CHO-bearing peaks in our reported CHO concentrations, we believe keeping "Total CHO" is most appropriate.
L134: See comments above and in our past response about SPE bias
L175: Spaces between units placed
L184: Spaces between units placed
L208-209: Spaces between units placed
L242-244: Reasons for CRAM evading detection by UF and SPE are size- and chemical-fractionation bias. We have added "due to size- and chemical-fractionation biases" to this sentence.
L253: We agree that the implied changes in projected CRAM ^{14}C-ages adds value to the paper.
L365-366: Subscript D2O has been added.
L372: Spaces between units placed

<u>Supplemental Information:</u>
L11: Change made
L45: Change made

L66-71: We have revised the text for clarity. It now reads: "*The similarity in PPL vs. total seawater [CRAM]:[TCHO] ratios suggests that SPE does not preferentially isolate CRAM over TCHO but seemingly isolates similar sub-fractions of both compound classes.*"
L82: Change made
L117-118: Changes made
L155-156: Changes made
L176: Changes made
L204-205: Changes made
L226-231: Changes made
L237-240: Changes made
Table S2: Changes made
Table S4: Changes made

Reviewer #3 (Remarks to the Author):

I still believe that the manuscript is novel and appropriate for publication in Nature Communications. I also appreciate the new information using SPE-DOM. However, I am still unsure as to the validity and error associated with the conversion to carbon content/concentrations given in the section on spectral integration of compound classes. The authors do state in their response that additional details are given, but without line numbers and track changes they are hard to follow - inspecting both the revised and the original manuscript I cannot find these details (now lines 402-424). This conversion appears to come from a paper on H and O content in phytoplankton (ref 43) using H/C ratios table S3. Are there not larger ranges in the H/C ratios for the compound classes reported elsewhere (or as FT ICR MS data may suggest)? Is H/C = 1 used for CRAM (Table S3)? I would a appreciate a bit more discussion on this point so we can know if the significant decreases in CRAM (3 umol C/kg) with depth (line 194) are truly significant, or just in the noise. Otherwise, I am satisfied with the review response and revisions.

We thank the reviewer for the valuable comments. We have provided a tracked changes document highlighting all changes made from the original manuscript file and have added more detail to the Supplementary Information.

Regarding the conversion of the H:C ratio, this is an excellent question. We have used an H:C ratio range of 0.8 to 1.4 based on the published FTICR-MS range for CRAM. The difference between the surface and deep ocean at each specific ratio ranged from 5.0 µmol C kg^{-1} to 2.0 µmol C kg^{-1}. The average CRAM concentration in the surface samples ranged from 31.4 to 22.5 µmol C kg^{-1} (average 26.3 µmol C kg^{-1}) and from 26.4 to 20.5 µmol C $kg^{-1}$for the deep samples (average 23.0 µmol C kg^{-1}). Using a two-sample t-test, we found that these two samples are significantly different (p = 0.05, t-value = 5.31).

Using the range of H:C ratios, we observed a decrease of 3.3 µmol C kg^{-1}, which is close to our 3.0 µmol C kg^{-1}using a specific CRAM H:C ratio of 1.0.

We avoid adopting a range of H:C ratios in our quantification for several reasons:

1. Using a range of H:C ratios would imply that we assign the same weight to each value within the range and assume that CRAM concentration is evenly distributed across the entire H:C range. We have no evidence to support this assumption, and given the chemistry of complex mixtures, it would not be valid.
2. Relying solely on the FTICR-MS range of H:C could introduce bias since FTICR-MS, in its current settings, is not a quantitative method. High intensity of specific mass features does not necessarily indicate higher concentrations compared to other mass features.

Instead, we chose a single H:C ratio of 1.0 for the following reasons:

1. It represents an approximate average of the proposed H:C range.
2. It reflects the common backbone structure of different CRAM isomers. According to the structure proposed by Hertkorn et al. (2006), the common backbone of the proposed

CRAM structure has an H:C ratio between 1.1 and 1.0, with different isomers varying by having additional side CH_3 group or an extra double bond.

3. This approach ensures that the sum of all %C percentages, including those from other major compounds (e.g. peptides, TCHO), does not exceed 100% C.

Regarding the other compound classes (e.g., peptides and carbohydrates), each of these classes is composed of known chemical structures with similar backbone configurations. Therefore, we adopted the single ratio provided by Anderson (1995) and further explained in Fox et al. (2018).

We have incorporated these points into the revised Supporting Information document.

Response to Reviewer Comments – McKee et al. 2024

We again thank the reviewers for their constructive and detailed comments. We have addressed the reviewers' comments and provide our responses (in blue) below, including information about the conversion of carbon content.

REVIEWER COMMENTS

Reviewer #3 (Remarks to the Author):

I am satisfied with author comments and recommend this article for publication. I only have one last minor comment:

Pg 8 line 13: why would CRAM be photochemically labile? Since CRAM does not likely absorb solar radiation, I would not expect it to be susceptible to direct photodegradation (perhaps indirect is possible however).

The discussion of additional possible CRAM cycling mechanisms (including photochemical oxidation, heterotrophy, priming, etc.) was specifically requested in the first round of review by Reviewer #1. From Hertkorn et al., 2006, some CRAM is hypothesized to have a few conjugated C=C bonds. This could allow light to absorb and possibly oxidization of CRAM functional groups. Indirect photochemical oxidation of CRAM through UV-induced hydroxyl radicals (from water) is also possible. There is substantial evidence that certain components within the CRAM pool can undergo photodegradation (e.g. Stubbins et al., 2010).

Reference

Stubbins, A., Spencer, R.G., Chen, H., Hatcher, P.G., Mopper, K., Hernes, P.J., Mwamba, V.L., Mangangu, A.M., Wabakanghanzi, J.N. and Six, J., 2010. Illuminated darkness: Molecular signatures of Congo River dissolved organic matter and its photochemical alteration as revealed by ultrahigh precision mass spectrometry. Limnology and Oceanography, 55(4), pp.1467-1477.
